# ER‐phagy: shaping up and destressing the endoplasmic reticulum

**DOI:** 10.1111/febs.14932

**Published:** 2019-06-10

**Authors:** Simon Wilkinson

**Affiliations:** ^1^ Edinburgh Cancer Research UK Centre MRC Institute of Genetics and Molecular Medicine University of Edinburgh UK

**Keywords:** ERLAD, ER‐phagy, FAM134B, microautophagy, recovER‐phagy

## Abstract

The endoplasmic reticulum (ER) network has central roles in metabolism and cellular organization. The ER undergoes dynamic alterations in morphology, molecular composition and functional specification. Remodelling of the network under fluctuating conditions enables the continual performance of ER functions and minimizes stress. Recent data have revealed that selective autophagy‐mediated degradation of ER fragments, or ER‐phagy, fundamentally contributes to this remodelling. This review provides a perspective on established views of selective autophagy, comparing these with emerging mechanisms of ER‐phagy and related processes. The text discusses the impact of ER‐phagy on the function of the ER‐ and the cell, both in normal physiology and when dysregulated within disease settings. Finally, unanswered questions regarding the mechanisms and significance of ER‐phagy are highlighted.

**Abbreviations**
AMPK: adenosine monophosphate‐activated kinase
ATF6: activating transcription factor 6
Atg11BR: Atg11‐binding region
ATG: autophagy‐related
ATL1‐3: Atlastins 1–3
ATZ: mutant α‐1‐antitrypsin
COPII: coat protein complex II
EGF: epidermal growth factor
eIF2α: eukaryotic initiation factor 2 alpha
ERAD: ER‐associated degradation
ER: endoplasmic reticulum
ERES: ER exit site
ERLAD: ER‐to‐lysosome–associated degradation
ESCRT: endosomal sorting complexes required for transport
FAM134B: family with sequence similarity 134 member B
FIP200: FAK‐interacting protein 200 kDa
FIR: FIP200‐binding region sequences
GIM: GABARAP‐interacting motif
GnRHR: gonadotrophin‐releasing hormone receptor
Grp78: 78 kDa glucose‐regulated protein
HSAN: hereditary sensory and autonomic neuropathy
HSP: hereditary spastic paraparesis
IRE1α: inositol‐requiring enzyme 1α
LAP: LC3‐associated phagocytosis
LD: lipid droplet
LIR: LC3‐interacting region
MAMs: mitochondria‐associated membranes, ER‐mitochondrion contact sites
MEFs: mouse embryonic fibroblasts
mTORC1: mammalian target of rapamycin complex 1
NBR1: neighbour of BRCA1
NDP52: nuclear dot protein 52
NE: nuclear envelope
NPC1: Niemman–Pick type C disease protein 1
OPTN: optineurin
PC: procollagen
PERK: protein kinase R (PKR)‐like endoplasmic reticulum kinase
pER: peripheral ER
PI3KC3 : class III phosphatidylinositol 3′‐kinase (VPS34)
PI3P: phosphatidylinositol‐3′‐phosphate
rER: rough ER
RETREG1: reticulophagy regulator 1
RHD: reticulon homology domain
RTN3: reticulon 3
sER: smooth ER
SQSTM1: sequestosome 1
STING: stimulator of interferon genes
STX17: syntaxin 17
TAX1BP1: TAX1‐binding protein 1
TBK1: TANK‐binding kinase 1
UBAN: Ub‐binding domain in ABIN proteins and NEMO
ULK: Unc51‐like kinase
UPR: unfolded protein response
VAMP8: vesicle‐associated membrane protein 8
WIPI2: WD repeat domain, phosphoinositide interacting 2 protein
XBP1: X‐box–binding protein 1

## Introduction

**Figure 1:**
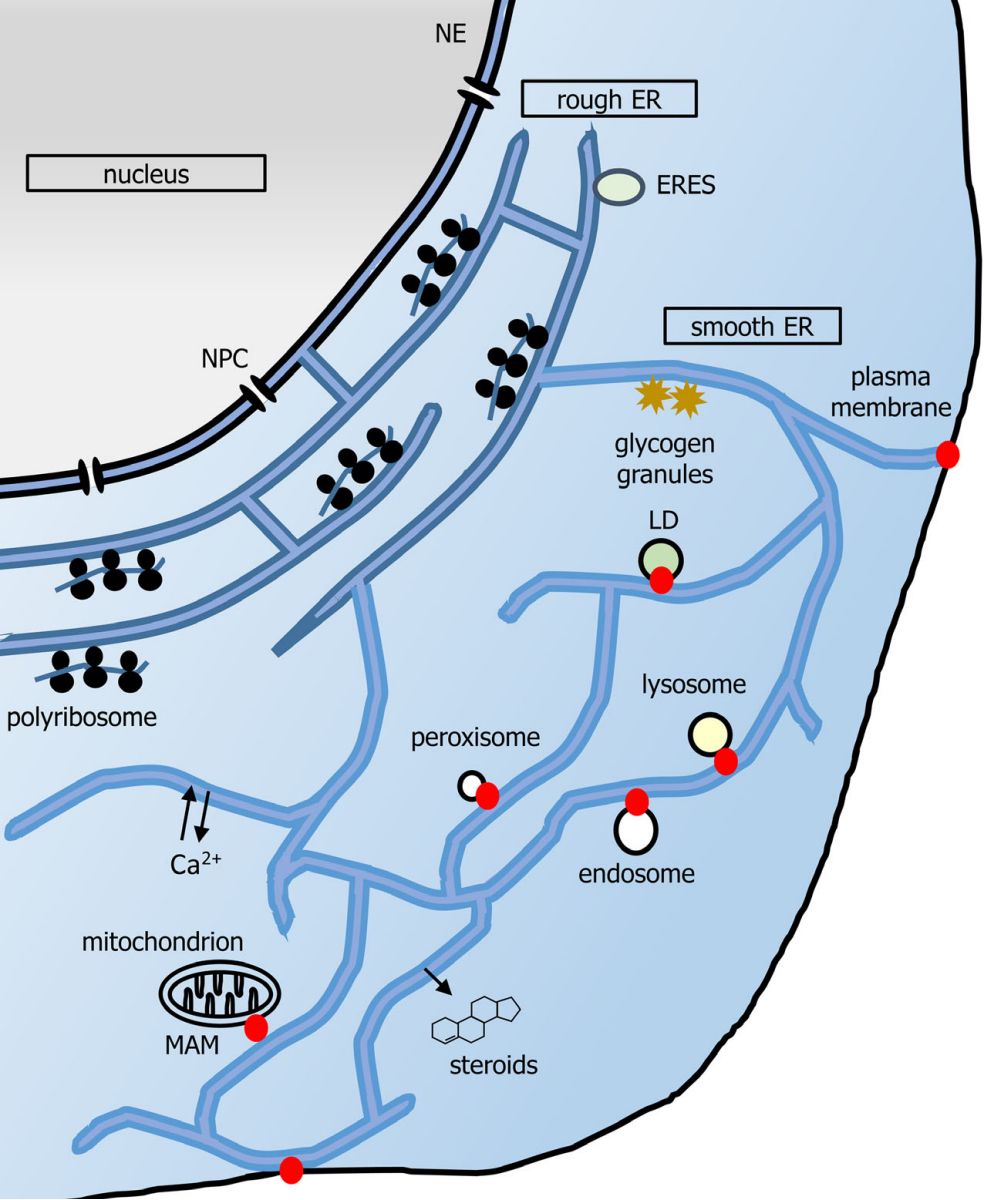
A schematic of mammalian ER. Nuclear pore complexes (NPCs) gate nucleocytoplasmic transport at the NE. The pER is composed of the rER and sER. rER (darker blue) is composed of flattened, stacked, frequently fenestrated, sheets, connected by helicoidal junctions (shown in cross section here). rER is studded with polyribosomes synthesizing secretory protein and functions in import, folding, glycosylation and onward secretion of such protein. Onward transport originates from ribosome‐free subdomains of rER (ER exit sites, ERESs). Smooth ER (sER, lighter blue) extends in a reticular network throughout the cell, characterized by three‐way junctions. ER tubules also exist in dense arrays in the perinuclear region (not shown for simplicity). The smooth ER functions in detoxification reactions, and lipid and steroid synthesis. Lipid synthesis contributes to organellar membrane generation, for example, during formation of LDs. Organelle contact sites (red dots) may also regulate signalling, for example, immune signalling and transfer of calcium to mitochondria both occur at MAMs. Contact sites may also regulate membrane dynamics, for example, endosome budding and mitochondrial fission. Although contact sites may be at the rER or sER, depending upon the organelle (e.g. both in the case of MAMs), for clarity they are depicted herein at the cell periphery. Note that the yeast ER has a different morphology; extensive cortical ER runs parallel to the plasma membrane, and is connected by tubules to the perinuclear ER, which delimits the nucleoplasm.

The endoplasmic reticulum (ER) is a ubiquitous subcellular compartment of eukaryotic cells. Mammalian ER is a continuous, lipid bilayer‐bound lumen. It is divisible into the nuclear envelope (NE) and a cytoplasmic peripheral ER (pER) composed of flat, sac‐like sheets and a reticulated, tubular network 1. The ER is a key organelle in support of metabolism and in control of subcellular organization and signalling (Fig. 1). Ribosome studded sheets (rough ER, rER) serve in the biosynthesis of transmembrane and secreted proteins. The oxidizing rER lumen facilitates disulphide bond formation within nascent polypeptides and contains enzymes that catalyse glycosylation. The tubular smooth ER (sER) functions in lipid and steroid hormone synthesis, and detoxification. The ER acts as a dynamic intracellular calcium (Ca^2+^) reservoir, controlling cytosolic calcium levels. Finally, the ER membrane houses junctional complexes at contacts with organelles including peroxisomes, lipid droplets (LDs), the Golgi apparatus, mitochondria, endosomes and the plasma membrane. These contacts regulate organellar function, Ca^2+^ homeostasis, lipid composition, fission, trafficking and participation in signal transduction events 2.

The ER undergoes dynamic alterations in morphology, molecular composition and functional specification. For instance, the ER is remodelled downstream of acute stimuli, such as compromise of ER protein or lipid metabolism. Indeed, the best‐described ER remodelling network is the unfolded protein response (UPR). In the UPR, lumenal unfolded protein binds the chaperone Grp78 (78 kDa glucose‐regulated protein), titrating this away from the transmembrane sensors PERK [protein kinase R (PKR)‐like endoplasmic reticulum kinase], IRE1α (inositol‐requiring enzyme 1 alpha) and ATF6 (activating transcription factor 6). Loss of Grp78 binding activates these sensors. The consequent cytosolic signalling cascades mediate restoration of ER status by cessation of general protein translation, via PERK‐mediated phosphorylation of the translation factor eIF2α (eukaryotic initiation factor 2 alpha), and by transcriptional upregulation of lumenal oxidoreductases and chaperones, and ERAD (ER‐associated degradation) factors 3. ERAD is the major proteasomal pathway for degradation of unwanted ER membrane or lumenal proteins 4. The ERAD machinery drives retrotranslocation of polypeptides to the cytosol, whereupon proteasomal‐mediated proteolysis occurs. However, acute ER remodelling is not always homeostatic. For example, upon Epidermal Growth Factor (EGF) stimulation, Reticulon 3 (RTN3) protein drives tubulation of pER and consequent ingress into the juxta‐plasma membrane region. This in turn facilitates EGF receptor endocytosis 5. ER remodelling can also fail or be overwhelmed in disease settings. Consequentially, the loss of vital ER functions leads directly to deterioration in cell health or, alternatively, unresolved UPR signalling promotes pathologic inflammation, cell death and even tumourigenicity 6, 7, 8. ER status is also specified by differentiation programs. For example, the sarcoplasmic reticulum has a prominent role in regulating cytosolic Ca^2+^ fluxes within skeletal muscle. Some highly secretory cells, such as pancreatic acinar cells, are majority composed of abundant, polarized rER; conversely, steroid hormone producing adrenal cells contain an extensive, specialized sER. ER status associated with differentiation state is also dependent upon gene expression and signalling networks. For example, the UPR transcription factor XBP1 (X‐box‐Binding Protein 1) drives expansion of rER during differentiation of antibody‐secreting plasma cells 9 and gastric chief cells 10.

**Figure 2:**
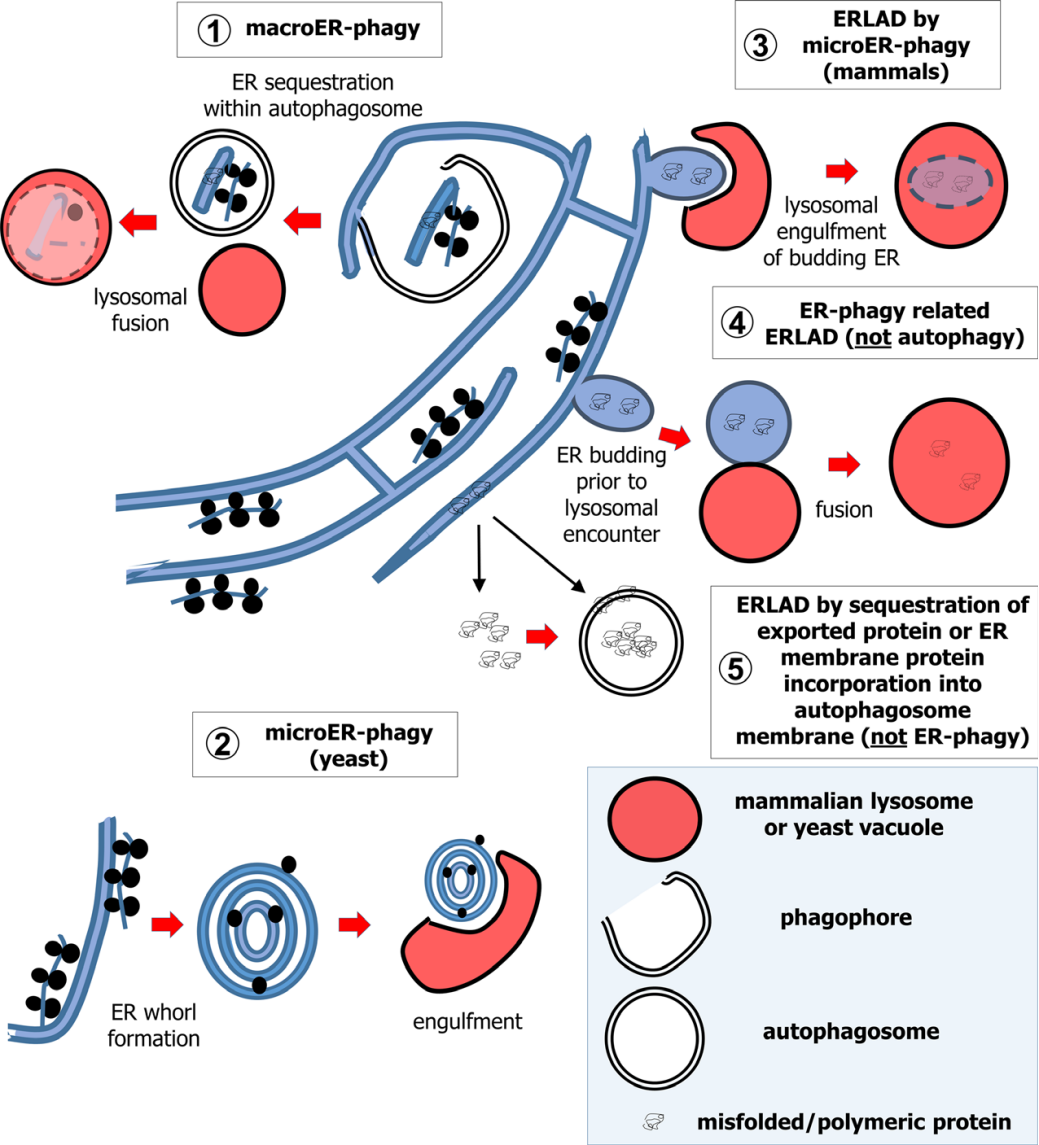
Pathways by which ER material transits to the lysosome. This review will reference five distinct routes via which ER fragments or lumenal material may be delivered to the lysosome. These include processes that are either *bona fide* ER‐phagy pathways, or related processes. Firstly, in macroautophagy (macroER‐phagy (1)), fragments of ER are sequestered by the growth of an encircling double‐membraned phagophore, which then forms an enclosed autophagosome and fuses with lysosomes. MacroER‐phagy can participate in ERLAD (ER‐to‐lysosome‐associated degradation) if particular proteasome‐resistant ER proteins are concentrated within the cargo fragment of ER. MicroER‐phagy is said to occur when lysosomal invagination or protrusion engulfs portions of ER. In yeast microER‐phagy (2), the ER expels whorls of membrane prior to vacuolar invagination. In mammals (3), procollagen‐enriched buds of ER forming from ER exit sites (ERESs) may be targeted in a microautophagy‐mediated ERLAD pathway. In contrast, non‐ER‐phagy processes that involve some or all of the core autophagy machinery are (4) an ER‐phagy–related ERLAD pathway in which single membrane ER‐derived vesicles packed with misfolded lumenal protein species, such as mutant α‐1‐antitryspin, fuse with lysosomes and (5) hypothetic autophagy‐dependent but non‐ER‐phagy ERLAD pathways, wherein aggregated or mutant protein would be expelled from the ER prior to cytosolic sequestration by autophagy or be incorporated directly from the ER membrane into the delimiting membrane of the autophagosome.

Recent findings have revealed that autophagy, the transport of cytoplasmic components into the lysosome for degradation, is a key ER remodelling process 11. Two main forms of autophagy regulate ER status. In microautophagy, sequestration of cytoplasmic material occurs via engulfment into endosomes or lysosomes 12. Conversely, macroautophagy sequesters cytoplasm in nascent phagocytic vesicles, called autophagosomes, which fuse with lysosomes 13. In some systems, ER perturbations upregulate macroautophagy in order to alleviate ER stress 14. Mechanistically, autophagy might indirectly regulate ER status. However, this review focuses on mechanisms by which the ER remodelling occurs by direct and selective degradation of ER in the lysosome. Most notably, this occurs via macroautophagy (macroER‐phagy) or microautophagy (microER‐phagy) of ER fragments, collectively ER‐phagy (Fig. 2). ER‐phagy‐related processes, such as lysosomal degradation of ER‐derived vesicles packed with ER lumenal content, also play a role; they will be compared with ER‐phagy herein (Fig. 2). Importantly, ER‐phagy responses – also termed reticulophagies 15‐ and related processes are emerging as mechanistically diverse and important players in ER remodelling; ER‐phagy has been observed in insect 16, plant 17, yeast and mammalian cells 11. This review will also demonstrate that such ER‐phagy also plays a key role in normal physiology and may be overwhelmed or aberrant in a number of disease conditions, including neurodegenerative disorders or cancer.

## Overview of autophagy

In order to frame our current knowledge of ER‐phagy, key general autophagy principles are outlined below. More can be found in dedicated review articles 12, 13, 18, 19. This review focuses on mammals. However, the text highlights other examples where informative.

### The core macroautophagy machinery

Macroautophagy is initiated via the co‐ordinated action of complexes of evolutionarily conserved ATG (Autophagy‐related) proteins, which results in the generation and expansion of nascent double lipid bilayer structures (phagophores or isolation membranes), which close around cytoplasmic material to form double‐membraned autophagosomes. Dynamic signal transduction regulates localization and activity of many ATG proteins in response to stimuli such as nutrient, ER or hypoxic stress. Basal macroautophagy also occurs in most systems, reflecting the autophagy activity permitted by tonic signalling in unchallenged cells or animals.

**Figure 3:**
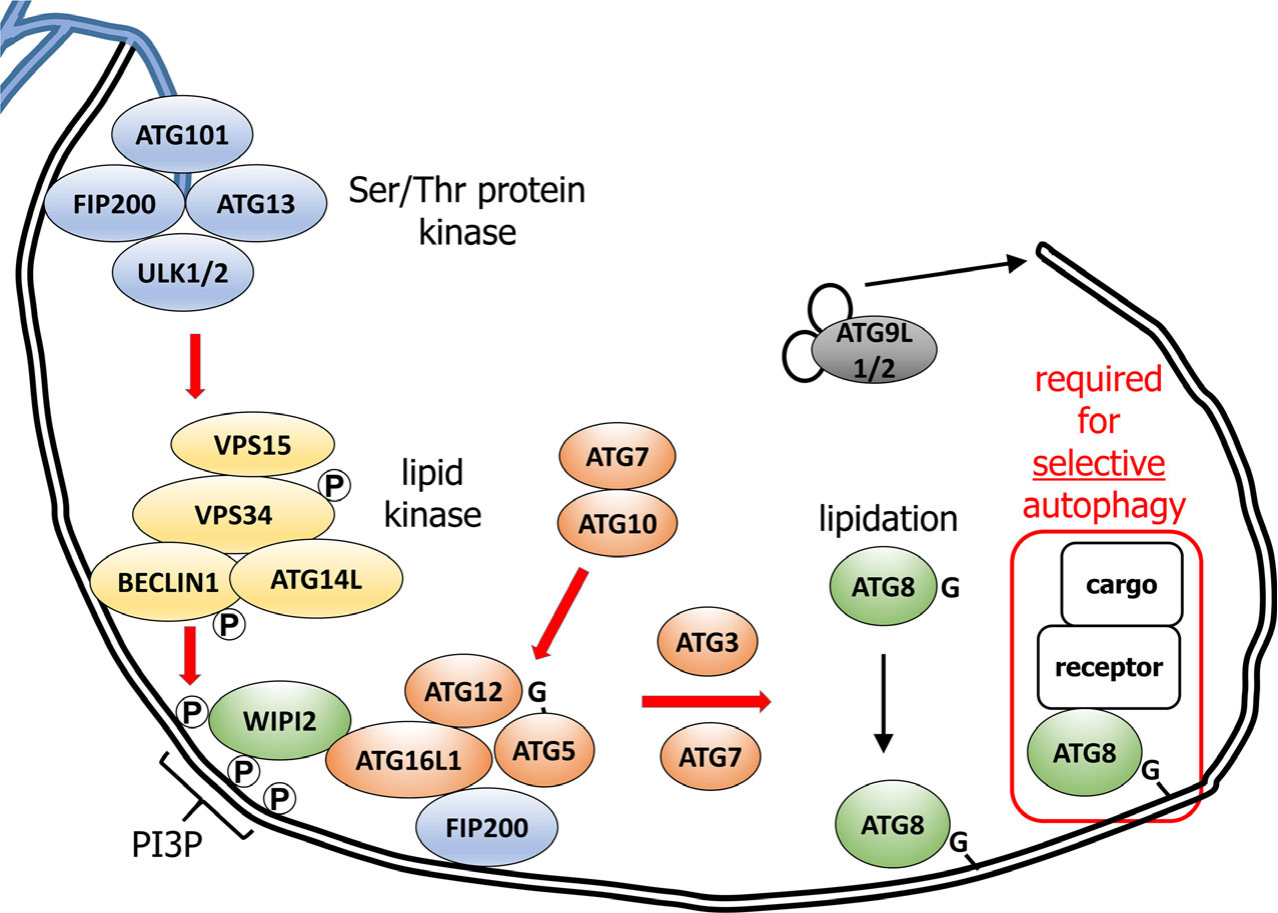
Essential mechanism of autophagosome generation in mammals. A phagophore is shown here (double black lines represent the dual lipid bilayer), notionally extending from an ER cradle (blue tubules). The hierarchy of ATG protein action that initiates and matures the phagophore is depicted as described in the text. Briefly, the ULK1/2 complex activity drives VPS34 complex‐mediated phosphorylation of phosphatidylinositol to phosphatidyl‐3′‐inositolphosphate (PI3P), which in turn recruits WIPI2. WIPI2 and FIP200 recruit the ATG5 complex. The ATG5 complex acts with ATG3 and ATG7 to attach phosphatidylethanolamine in the phagophore to the exposed C‐terminal glycine of proteolytically processed LC3/GABARAP. Further lipid is delivered from various sources, such as tubular endosomes; the transmembrane ATG proteins ATG9L1/2 co‐ordinate this. Note that while LC3/GABARAP plays a role in accelerating expansion and closure of phagophores, it is also required for selection of cargo via interaction with cargo receptors.

Autophagy protein complexes act in a temporal hierarchy (Fig. 3). The ULK complex is an early‐acting assembly, comprising the scaffolding ATG proteins FIP200 (FAK‐interacting Protein 200 kDa, alias RB1CC1), ATG13, ATG101 and the serine–threonine protein kinases ULK1/2 (Unc51‐like Kinases 1/2) 20. The enzymatic activities of ULK1/2 promote autophagy and are key signal integrators; phosphorylations of ULK1 by mTORC1 (Mammalian Target of Rapamycin Complex 1) and AMPK (Adenosine Monophosphate‐activated Kinase) inhibit and activate kinase activity, respectively 21. Upon ULK1/2 activation, the ULK and VPS34 complexes (discussed below) recruit to nascent phagophores, which are generated via deformation, budding and fusion of mixed membrane sources, including endosomes, plasma membrane and the ER 19. Indeed, the phagophore membrane may be contiguous with the ER (Fig. 3), although this does not prove that the lipids therein are derived predominantly from the ER 22, 23. In either case, the relatively small lipid and protein mass that could potentially exit the ER via this route is not considered selective ER‐phagy.

Phosphatidylinositol‐3′‐phosphate (PI3P) lipid is generated from phosphatidylinositol (PI) at the phagophore by the action of the Class III Phosphatidylinositol 3′‐Kinase (VPS34) complex (PI3KC3 complex I). ULK1/2 can phosphorylate two components of this VPS34 complex, the VPS34 lipid kinase and BECLIN1 (ATG6). Other complex members include VPS15 and ATG14L, the latter of which targets the complex to the phagophore. PI3P generated thusly at the phagophore recruits the lipid‐binding protein WIPI2 (WD Repeat Domain, phosphoinositide‐interacting 2 protein) 24. ULK1/2 may also stimulate ATG9L1/2 to deliver vesicular membrane to growing phagophores 26, 27. Both WIPI2 and FIP200 interact with ATG16L1 to promote recruitment of the ATG5‐12 (ATG16L1‐ATG5‐ATG12) complex 28, 29. ATG5 is covalently modified by ATG12 in a ubiquitin‐like conjugation reaction, catalysed by ATG7 and ATG10. The ATG5‐12 complex acts as an E3‐like enzyme in a second ubiquitin‐like conjugation reaction called lipidation, in partnership with ATG7 and ATG3 (E1‐ and E2‐like activities). In this reaction, ubiquitin‐like proteins of the mammalian LC3/GABARAP (ATG8) family covalently modify phosphatidylethanolamine in the phagophore, enhancing expansion and closure. Note that, in humans, LC3/GABARAP proteins are divided into the MAP1LC3 (LC3A, LCB and LC3C) and GABARAP (GABARAP, GABARAPL1 and GABARAPL2/GATE16) subfamilies, whereas in yeast a single orthologue termed Atg8 exists.

Some stimuli engage noncanonical forms of macroautophagy that are independent of some core ATG proteins, such as BECLIN1 or ULK1/2 30. Outwith autophagy *per se,* there are macroautophagy‐related membrane trafficking processes that similarly depend upon a subset of ATG proteins. For example, LC3‐associated phagocytosis (LAP) is the ULK complex‐independent modification of plasma membrane‐derived phagosomes with LC3/GABARAP 31. There is evidence for the existence of ER‐phagy–related ER degradation pathways that may exhibit similarly unconventional features, as described later.

### Selective macroautophagy is defined by cargo recognition

Autophagosome generation from the ER can result in adjacent ER fragment capture via simple spatial proximity 22, 23. However, additional molecular determinants, other than ATG proteins, are required for efficient selective sequestration of ER 34. Instructively, mature research on other selective macroautophagy processes such as mitophagy (mitochondrial cargo), aggrephagy (protein aggregates) and xenophagy (cytosolic pathogens), has revealed a ubiquitous requirement of cargo receptor proteins (Fig. 3), which molecularly bridge the autophagosome and the cargo 35. In mammals, this frequently involves direct recognition of both polyubiquitin modifications of cargo and of LC3/GABARAP, via discrete regions of the receptor. This is exemplified by the receptors p62/SQSTM1 (Sequestosome 1), OPTN (Optineurin), NDP52/CALCOCO2 (Nuclear Dot Protein 52), TAX1BP1/CALCOCO3 (TAX1‐binding Protein 1) and NBR1 (Neighbour of BRCA1). Notably, linear peptide motif(s) with the minimal consensus sequence (W/Y/F)_1_‐X_2_‐X_3_‐(L/I/V)_4_, known as LC3‐interacting regions (LIRs) 36 or, a subset of the former, GABARAP‐interacting motifs (GIMs) 37, mediate receptor interaction with LC3/GABARAP. Yeast cargo receptors may also bind Atg11, which has no mammalian orthologue 38. Cargo receptors may also integrate signals to moderate selective autophagy. For example, phosphorylation of OPTN stimulates ATG8 and ubiquitin binding 40.

### Selective microautophagy processes

In microautophagy, endosomes or lysosomes (the vacuole in yeast), can invaginate to subsume cargo. Alternatively, lysosome or vacuole membranes can protrude to enwrap cargo 12, 41. Microautophagy pathways employ diverse molecular mechanisms. Nonetheless, the dual mechanistic principles of selective macroautophagy – membrane remodelling and recognition of cargo – apply to selective microautophagy. Microautophagy of peroxisomes in the yeast *Pichia pastoris* exemplifies this. Proteins such as Atg18 42 and Vac8 43, 44 drive vacuolar membrane protrusion while the core Atg proteins adjacently build an Atg8‐labelled phagophore‐like structure that donates membrane to the protrusions 45. Peroxisomal Atg30 acts as a receptor, linking peroxisomes to Atg11 on the vacuole and phagophore 46. There are few molecular details on mammalian microautophagy, with the partial exception of the endosomal invagination pathway 47. This process is characterized by the use of endosomal sorting complexes required for transport (ESCRT)‐family proteins for membrane remodelling, as are some yeast microautophagy pathways 48. Recognition of cargo for internalization is mediated by the chaperone Hsc70 47; in fission yeast, a similar process may involve Nbr1 49. Intriguingly, the mammalian orthologue NBR1 is a macroautophagy receptor that can also be degraded by endosomal microautophagy 50.

## ER‐phagy pathways: mechanisms and importance

Macroautophagy of the ER (macroER‐phagy) was first identified ultrastructurally 16, 51, 52. For instance, autophagosomes packed with ER fragments were seen in cultured hepatocytes recovering from phenobarbital‐induced sER expansion *in vitro* 51 or in guinea pig pancreata after subcutaneous cobalt injection 52. In the first description of microER‐phagy, induction of the UPR in the yeast *Saccharomyces cerevisiae* was seen to drive ER expansion, resulting in a counterbalancing expulsion of concentric whorls of ER membrane, which were then engulfed by vacuolar invagination, all of this occurring independently of Atg proteins 53, 54 (Fig. 2). However, no ER‐phagy–specific molecular players were identified in the above systems, thereby limiting investigation of mechanism and of functional importance. Conversely, core macroautophagy proteins have also been shown to regulate ER size and function. For example, ER stress triggers prosurvival macroautophagy in mouse embryonic fibroblasts (MEFs) 14. Tissue‐specific deletion of murine *Atg5* in terminally differentiated B‐lymphocytes (plasma cells) 55, or *Atg5* or *Atg7* in pancreatic acinar cells, drives ER expansion, UPR and cell death 56. *Atg5* deletion in mature T‐lymphocytes results in ER accumulation and defective Ca^2+^ signalling 58. Finally, *Atg7* is required for the secretion of collagen from mouse chondrocytes, an important process in bone growth. In the absence of this, procollagen II accumulates within a distended ER 59. It is possible that some of these phenomena involve selective ER degradation, but the lack of known ER‐phagy–specific genes available to test during the execution of these studies precluded determination of this. However, recent breakthroughs have identified several ER membrane‐resident cargo receptors that specifically facilitate ER‐phagy, enabling rapid progress in establishing mechanistic and functional principles. Thus, the following exploration of ER‐phagy is structured around a discussion of individual receptors, highlighting the following principles: selectivity determinants for autophagy‐mediated recognition of the ER *per se* and of particular subcomponents thereof; fragmentation of ER to facilitate sequestration; co‐ordination of ER‐phagy via cell signalling.

### FAM134B and Atlastins in sheet turnover and proteostasis

**Figure 4:**
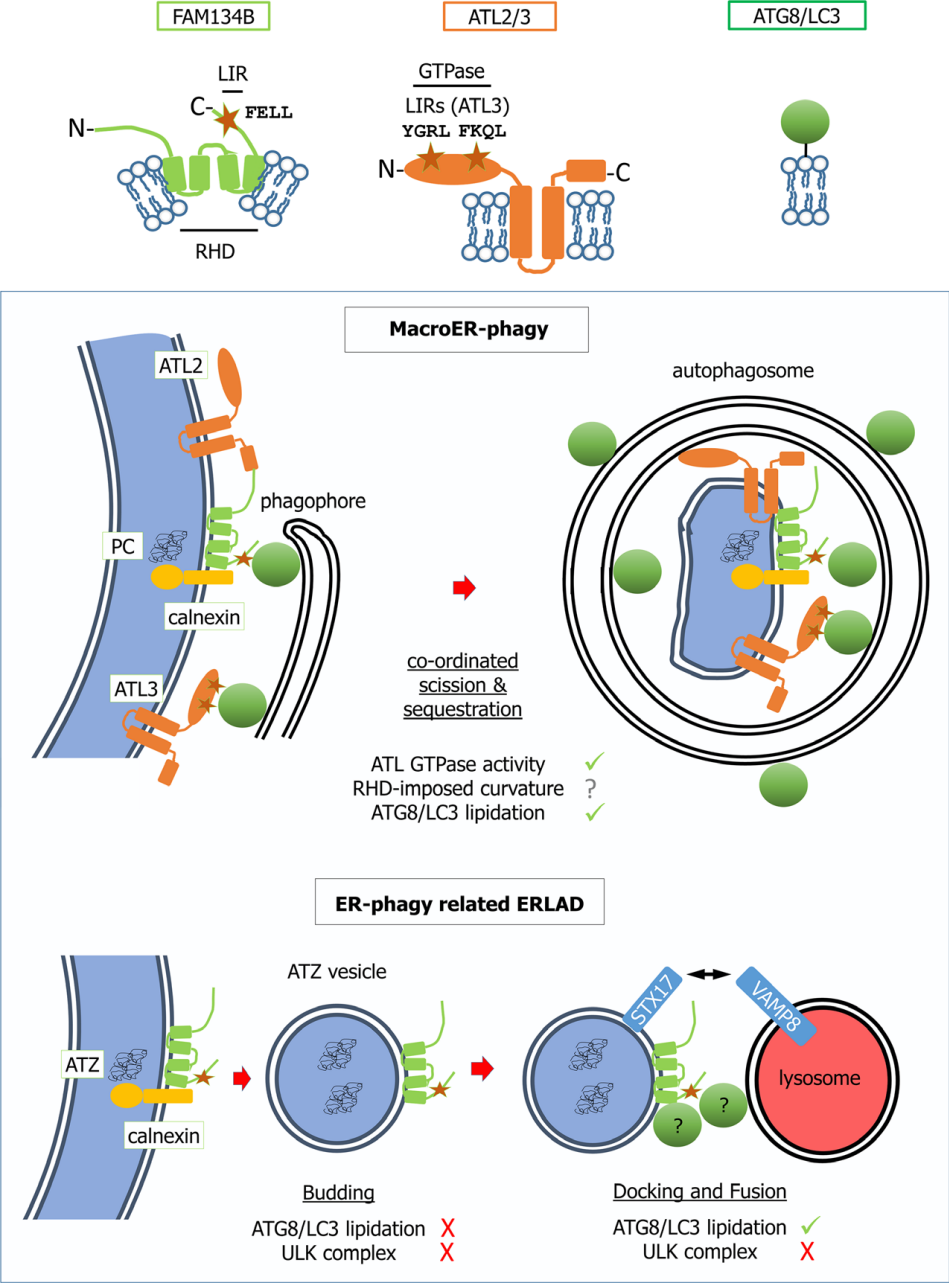
A model of FAM134B and Atlastin function in delivery of ER content to lysosomes. In the key at the top of the diagram, the core sequence of each LIR motif is shown for each receptor (RHD, reticulon homology domain; GTPase, dynamin‐like GTPase domain). The box provides a schematic overview of FAM134B‐ and Atlastin‐dependent macroER‐phagy and of ER‐phagy‐related ERLAD of mutant α‐1‐antitrypsin (ATZ). Note that the cartoon of macroER‐phagy is shown particularly in the context of clearance of specific ER lumenal moieties (procollagen, PC), to illustrate the full breadth of our knowledge of this process, but macroER‐phagy likely operates in other contexts to functionally remodel the ER in different ways. In macroER‐phagy, the LIR motif of FAM134B drives clustering at sites of autophagosome genesis, probably aided by initial phagophore generation and recruitment of lipidated LC3/GABARAP. RHD‐mediated curvature in conjunction with the GTPase activity of the Atlastins (ATL2 and ATL3 depicted here), results in ER fragmentation. For tubular ER degradation (which is largely FAM134B‐independent, but RTN3L dependent) fragmentation may also be promoted by LC3/GABARAP‐mediated recruitment of ATL3 via LIR motifs (also known GIM motifs due to selectivity for GABARAP subfamily proteins). LC3/GABARAP‐mediated recognition of FAM134B (and ATL3 for tubular ER) also ensures that the ER fragment is incorporated into the mature autophagosome. In contrast, in ER‐phagy–related ERLAD, single‐membraned vesicles derive from the ER, incorporating FAM134B. However, interaction of FAM134B with LC3/GABARAP is only required for lysosomal fusion, along with the SNARE pairing of STX17 and VAMP8. This delivers the ER lumenal contents into the lysosome, although the membrane is donated to the lysosome, whereas in macroautophagy the entire fragment of ER, membrane and lumen, is internalized and degraded. A minimal LC3/GABARAP lipidation machinery, excluding ATG proteins such as the ULK complex, is required for ER‐phagy–related ERLAD. Selectivity for lumenal content in macroER‐phagy or ER‐phagy–related ERLAD is at least partly mediated via binding of FAM134B to the chaperone calnexin, which can in turn bind to misfolded or polymerized PC or ATZ.

Family With Sequence Similarity 134 Member B (FAM134B), also known as Reticulophagy Regulator 1 (RETREG1), is an ER membrane protein that preferentially localizes to ER sheets 33(Fig. 4). Post‐translational insertion into the lipid bilayer is mediated by an Reticulon Homology Domain (RHD), structurally defined by two hydrophobic hairpin helices that do not intrude into the ER lumen. The sequences N‐terminal and C‐terminal to the RHD are cytosolic, enabling a C‐terminal LIR motif (FELL) to mediate LC3/GABARAP recognition 33. FAM134B expression in human U2OS osteosarcoma cells causes ER fragmentation and coalescence into FAM134B and LC3/GABARAP‐enriched autophagosomes, dependent upon the LIR motif. Conversely, RNA interference (RNAi) against *FAM134B* in U2OS, or *Fam134b* knockout in MEFs, promotes ER expansion 33. Thus, FAM134B mediates basal macroER‐phagy. Nutrient starvation upregulates FAM134B‐dependent macroER‐phagy further. This occurs along with FAM134B‐independent LC3/GABARAP lipidation and turnover of p62/SQSTM1, highlighting FAM134B's ER‐phagy selectivity. Consistent with its sub‐ER localization, FAM134B predominantly acts upon ER sheets but not tubules 34.

How does FAM134B expression drive fragmentation of the ER and is this required for ER‐phagy? The asymmetric insertion of RHD domains into lipid bilayers causes membrane curvature, potentially facilitating scission 60. However, the extent to which the RHD domain of FAM134B contributes to fragmentation remains to be formally tested. Recently, Atlastins 1–3 (ATL1‐3) were identified as requirements for macroER‐phagy 61. Atlastins are dynamin‐superfamily GTPases that are anchored in the ER via two transmembrane helices (Fig. 4). The cytosolic GTPase activity drives homotypic ER membrane fusion and thus ER branching or scission. ATL2 binds to FAM134B, localizes with FAM134B at autophagosome biogenesis sites, and is required for FAM134B‐driven ER‐phagy. This observation strongly supports a role for receptor‐coordinated membrane fragmentation in ER‐phagy 61.

How does FAM134B determine ER status? FAM134B might indiscriminately target ER, merely to control organellar volume. Alternatively, ER‐phagy could also have more finely tuned actions upon the ER. One clear functional role emerging for FAM134B is in proteostasis 63. ER lumenal procollagen (PC) transits to the Golgi apparatus via Coat Protein Complex II (COPII)‐dependent transport from ER exit sites (ERES). However, data from human Saos‐2 osteosarcoma cells and MEFs indicate that some newly synthesized PC misfolds and is eliminated by FAM134B‐driven ER‐phagy (Fig. 4). Mechanistically, FAM134B binds the transmembrane protein calnexin, which recognizes unfolded PC via its lumenal chaperone domain 62. Whether FAM134B also assists in nucleation of PC aggregates or if FAM134B is recruited to ER subregions where PC‐calnexin has already clustered is unclear. Not just lumenal misfolded proteins but also ER transmembrane proteins such as mutant NPC1 (Niemman–Pick type C disease protein 1), may be subject to FAM134B‐driven, ER‐phagy–mediated sequestration. Mutant NPC1 degradation by ER‐phagy may be a compensatory pathway for ERAD 64. Taken together, these studies show that subregions of ER may be targeted by ER‐phagy receptors via recognition of specific ER moieties.

Roles for FAM134B in proteostasis may extend beyond canonical selective macroautophagy. For example, calnexin cooperates with FAM134B in ER removal of a polymerization‐prone, hereditary mutant of α‐1‐antitrypsin (ATZ) 63. Single ER membrane‐delimited vesicles form from sites of lumenal calnexin‐ATZ clustering. However, while FAM134B is incorporated into vesicles, the vesiculation process itself is FAM134B‐ and ATG independent. Nonetheless, partially reminiscent of LC3‐associated phagocytosis (LAP), LC3/GABARAP lipidation is required for vesicle fusion with endolysosomes. Fusion also relies upon the interaction between the ER soluble N‐ethylmaleimide‐sensitive factor attachment protein receptor (SNARE) protein Syntaxin 17 (STX17) and the lysosomal SNARE protein Vesicle‐Associated Membrane Protein 8 (VAMP8) 63. FAM134B‐LC3/GABARAP interaction at vesicle‐lysosome contact sites drives fusion, suggesting that lysosomal LC3/GABARAP recognizes vesicular receptor. However, this concept requires experimental confirmation (Fig. 4). This ER‐phagy–related process was termed a form of autophagy‐related ER‐to‐lysosome‐associated degradation (ERLAD) 63. Interestingly, an independent study showed that PC aggregates enter ER buds at ERESs 65. This occurs concomitant with LC3/GABARAP labelling of buds, and engulfment by lysosomal invagination and microautophagy. The molecular dependencies of this are unclear but, speculatively, they might have overlapped with the autophagy‐related mechanism of ERLAD described for ATZ. Importantly, ERLAD was more latterly suggested to be a useful overarching term for all processes that result in lysosomal clearance of faulty ER gene products that are proteasome resistant and escape ERAD, including some forms of *bona fide* ER‐phagy 62. For clarity, this is how the term shall be used in this review (Fig. 2).

Atg40, an ER‐phagy receptor in *S. cerevisiae*, is similarly organized to FAM134B, with an RHD and a single, C‐terminal Atg8‐interacting motif 66. Such cross‐species conservation of ER‐phagy illustrates its fundamental importance. Indeed, FAM134B function is important for cellular health. In MEFs and human A549 lung cancer cells, FAM134B protects against ER stressors; murine *Fam134b* knockout leads to ER dilation and cell death of peripheral sensory neurons 33. This reflects a form of hereditary sensory and autonomic neuropathy (HSAN type II) in humans caused by *FAM134B* nonsense mutations 67. However, it remains to be determined whether the primary cause of these neuropathies is defective FAM134B‐mediated procollagen (PC) quality control within the ER lumen, as might be suggested by the *in vitro* study described above 62. No effect of *Fam134b* knockout was reported in other organs, albeit in unchallenged mice. However, FAM134B's paralogues, FAM134A and FAM134C, also bind LC3/GABARAP 33. Their roles require investigation.

### RTN3L in tubular remodelling

**Figure 5:**
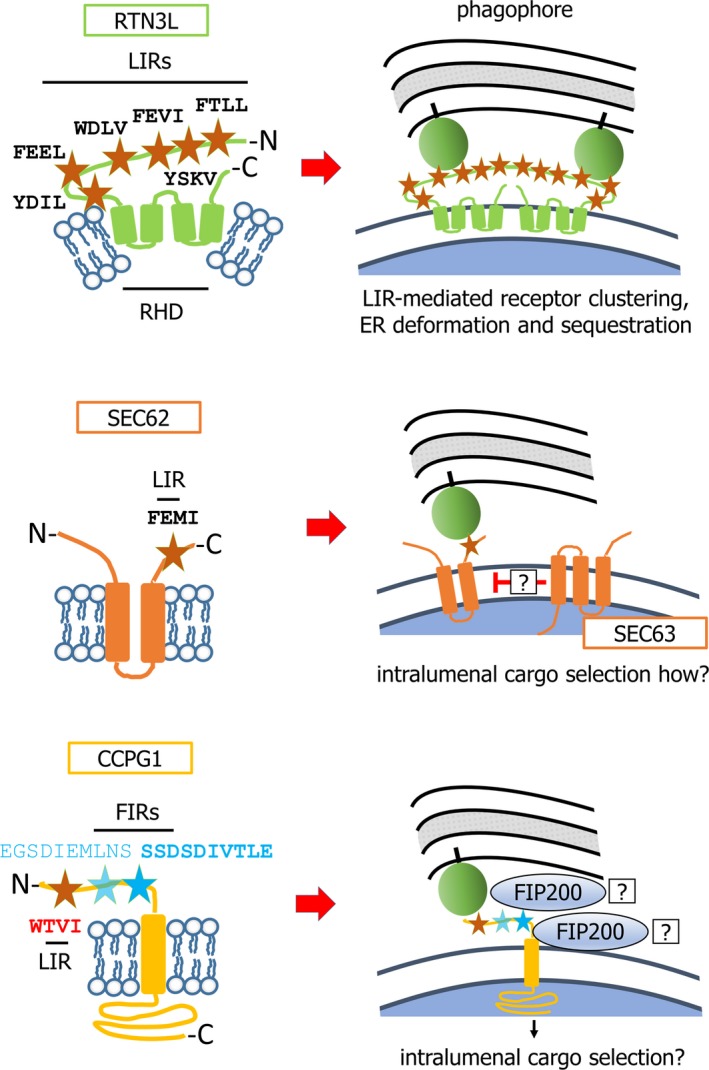
Models of action of RTN3L, SEC62 and CCPG1 in macroER‐phagy. Schematics of receptors RTN3L, SEC62 and CCPG1 are depicted on the left‐hand side of the diagram. Core LIR motif sequences are shown. Additionally, in CCPG1, minor (light blue) and major (bold blue) sequences supporting FIP200‐binding activity are shown (FIR = FIP200 interacting region). LC3/GABARAP molecules (green circles) bind LIR motifs in RTN3L to mediate focal recruitment at the nascent phagophore and oligomer formation, promoting ER curvature and incorporation of the eventual ER fragment into the mature autophagosome. The LIR motif of SEC62 mediates binding of ER fragments containing UPR‐upregulated proteins to the nascent phagophore. How these subregions of ER are generated or how SEC62 recognizes specific lumenal cargoes is unknown. When the UPR is at basal levels, SEC63 may bind SEC62 and compete for LC3/GABARAP interaction. Finally, CCPG1 uses a LIR to bind LC3/GABARAP on the phagophore and FIR regions to bind FIP200 on either the ER or the phagophore. Both interactions are required for sequestration of CCPG1‐enriched ER into autophagosomes. CCPG1 has a substantial (> 450 amino acid) lumenal domain that could hypothetically participate in recognition of specific lumenal cargoes.

Reticulons 1–4 (RTN1–4) are RHD‐containing ER‐reshaping proteins. RTN3 drives ER tubulation, sheet edge curvature and sheet fenestration 68, 69. However, the RTN3L splice isoform of RTN3 also has an extended cytosolic N terminus containing six LIR motifs 34. Using similar experimental approaches to the FAM134B study 33, it was demonstrated that RTN3L was a *bona fide*, LC3/GABARAP family‐binding macroER‐phagy receptor in nutrient‐starved MEFs (Fig. 5). In this context, RTN3L mediates degradation of tubular but not sheet ER. Importantly, as with FAM134B, RTN3L does not mediate LC3 lipidation or p62/SQSTM1 degradation, indicating a selective role in ER degradation and not in general autophagy responses. GABARAP interaction is required for fragmentation of the ER by focal clustering of multimerized RTN3L molecules 34. A requirement of the FAM134B LIR for fragmentation of ER was similarly seen, but explored in less depth, in a prior study 33. These observations underscore that the LC3/GABARAP lipidation machinery in ER‐phagy not only promotes phagophore growth and recognition of cargo but may also recruit activities required for ER membrane dynamics. It is unknown if Atlastins co‐operate with RTN3L in driving fragmentation and ER‐phagy. Functionally, initial RNAi data highlight a potential role for RTN3 in PC proteostasis 62. However, *Rtn3* null mice have no ER dysfunction phenotype 70. Given that heterodimers of RTN3L with shorter RTN3 isoform(s) are impaired in ER fragmentation 34, an *Rtn3l*‐specific loss‐of‐function mouse might yet reveal its physiological function.

### ATL3 as a GABARAP‐binding receptor

Although ATL2 cooperates with FAM134B in ER remodelling, the Atlastin ATL3 additionally contains two GABARAP‐selective LIR motifs (GIM motif) 71. These mediate ATL3 degradation by autophagy, and ER‐phagy of tubular ER (Fig. 4). The situation with ATL3 thus parallels RTN3L, where a known ER‐reshaping factor also acts as a receptor, potentially combining ER recognition and membrane‐reshaping principles. A point mutation within the GIM of *ATL3* that precludes GABARAP‐binding was found in the neuropathy HSAN type I 71, suggesting that dysfunctional ER‐phagy is part of the disease mechanism. Interestingly, loss‐of‐function of human *ATL1*, which also participates in ER‐phagy (discussed above), underlies a related degenerative condition of the central nervous system termed hereditary spastic paraparesis (HSP) 72.

### SEC62 and the UPR

SEC62 and SEC63 are ER transmembrane proteins that bind the SEC61 translocon to promote post‐translational entry of polypeptides into the rER. Mammalian (not yeast) SEC62 contains a cytosolic LIR motif at the C terminus, which plays no role in translocation 73. SEC62 specifically appears to mediate macroER‐phagy during cellular recovery from an acute UPR response (Fig. 5). Indeed, SEC62 does not participate in FAM134B‐driven PC proteostasis 62. SEC62‐dependent autophagosomes contain selected UPR‐upregulated proteins, including chaperones such as calnexins, but largely exclude other ER components, for example, ERAD proteins. This observation highlights once again the emerging theme of ER‐phagy–mediated recognition of specific subregions or subcompositions of ER. The molecular mechanism by which SEC62 facilitates ER‐phagy of specific intralumenal cargo requires investigation. It is not known how ER fragmentation occurs during SEC62‐mediated ER‐phagy. It is also unclear how this pathway is stimulated by the UPR. However, some evidence supports a model where SEC63 competes with mammalian LC3/GABARAP for SEC62 binding and thus inhibits ER‐phagy 73; the SEC62–SEC63 interaction might be lost during recovery from ER stress. The physiological function of SEC62‐mediated ER‐phagy at organismal level also requires investigation.

### CCPG1 in exocrine secretory cells

CCPG1 is a type II, single‐pass transmembrane protein 74. In contrast to FAM134B and RTN3L, CCPG1 contains a lumenal region of undefined function. The N‐terminal cytosolic region contains a LIR motif that promotes incorporation into autophagosomes. CCPG1 stimulates ER‐phagy upon overexpression in HeLa cells, dependent upon the LIR motif 75 (Fig. 5). Showing that endogenous CCPG1 is required for ER‐phagy, it was observed that *CCPG1* deletion blocked ER‐phagy in response to nutrient starvation, as seen previously for FAM134B or RTN3 deletion (see above). It is unclear to what extent complete nutrient starvation models physiological stimuli for ER‐phagy. It is a useful experimental tool to stimulate ER‐phagy in cultured cells and co‐opt the function of ER‐phagy receptors for mechanistic studies; however, these data should not be taken as suggesting that CCPG1 co‐operates with FAM134B or RTN3L in degradation of sheet‐like or tubular ER under physiologic conditions. This remains to be addressed. In this regard, endogenous CCPG1 loss also blocked ER‐phagy induced by the ER stressor DTT (dithiothreitol, an inhibitor of disulphide bond formation and protein folding), suggesting a link with acute ER stress responses (rather than recovery, as seen with SEC62). *CCPG1* has no sequence orthologue outside vertebrates. However, several features of CCPG1 function highlight similarities with *S. cerevisiae* Atg39, a receptor for autophagy of the perinuclear ER (equivalent to the mammalian NE). Atg39 is a single‐pass transmembrane protein with a cytosolic, N‐terminal Atg8‐interacting motif 66. Atg39‐driven ER‐phagy also requires an Atg11‐binding region (Atg11BR). Intriguingly, CCPG1 action in ER‐phagy also requires two FIP200‐binding region (FIR) sequences, similar to the yeast Atg11BR consensus, which bind the C‐terminal region of FIP200. This region of FIP200 is itself homologous to the C‐terminal coiled‐coil of yeast Atg11 that recognizes Atg11BRs 38. Finally, *CCPG1* transcriptional upregulation is triggered by the UPR 76, consistent with its role in DTT‐driven ER‐phagy. This provides an example of how ER‐phagy may be regulated by signal transduction, in this instance coordinating CCPG1‐dependent events with other transcriptionally induced ER remodelling activities.

The following questions arise regarding CCPG1‐driven autophagy mechanisms. What molecules provide ER membrane fragmentation activity? When does FIP200 binding occur? Prior to LC3/GABARAP lipidation and attachment to the phagophore? And for what purpose? One hypothesis states that FIP200 clusters with CCPG1 on the ER to mark sites of autophagosome biogenesis, prior to lipidation of LC3/GABARAP on the phagophore and ‘handover’ of CCPG1 (Fig. 5). Are there determinants for subER selectivity in CCPG1‐mediated ER degradation? RNAi data from Saos‐2 cells do show that CCPG1 may have minor roles in PC clearance 62. Indeed, when *Ccpg1* function is ablated in mice, exocrine pancreatic acinar and gastric chief cells display ER expansion 76. In particular, CCPG1‐deficient ER in the pancreas harbours numerous lumenal protein inclusions, resulting in UPR elevation. Mice remain viable under unchallenged conditions, but may be sensitive to proinflammatory stimuli during ageing. This requires further investigation. It has been speculated that CCPG1 and ER‐phagy act to directly remove these lumenal protein aggregates from the pancreas, but this idea also requires testing 77. Overall, CCPG1 exemplifies how ER‐phagy might have specific roles in determination of ER status in cell types that have specialized ER function.

### TEX264 and nutrient starvation

Very recently, TEX264 was identified as a single pass, transmembrane receptor for nutrient starvation‐induced ER‐phagy. It has a C‐terminal cytosolic region with a single LIR motif 78, 79. TEX264 is ubiquitously turned over by autophagy *in vivo* and is responsible for more than half of the nutrient starvation‐induced autophagic flux from the ER in cultured cells. Interestingly, not all ER proteins are equally sensitive to the presence of TEX264 for degradation by ER‐phagy, again suggesting mechanisms of selectivity related to the site of initiation of ER‐phagy via a particular receptor, or intrinsic recognition of select ER species via molecular interactions with the receptor. Time‐resolved imaging of TEX264 incorporation into ER foci within autophagosomes showed that LC3 recruitment preceded TEX264 recruitment at three‐way junction sites in the tubular ER. Interestingly, rings of TEX264 colocalizing with LC3 were produced upon recruitment of the former, suggesting that fragmentation of ER might be a late event, only occurring once loops of tubular ER are bound in close apposition to the membrane of an unclosed, but otherwise fairly complete, autophagosome.

### Other potential ER‐phagy receptors

The cytosolic cargo receptor p62/SQSTM1 recruits to ER‐containing autophagosomes and facilitates excess ER turnover in mouse liver 80. The ER transmembrane protein and UPR transducer IRE1α (Inositol‐requiring Enzyme 1α) binds p62/SQSTM1, as well as the other cytosolic ubiquitin‐binding receptors Optineurin and NBR1. This observation has led to the suggestion that ER‐phagy could sequester active IRE1α‐enriched ER subdomains in order to terminate UPR signalling 81. The proposed involvement of these ubiquitin‐binding receptors in ER‐phagy highlights a need to explore potential cytosolic ubiquitylation of ER membrane proteins in marking sites of ER‐phagy. Interestingly, ERES‐derived buds that are targeted by mammalian microER‐phagy were found to be labelled with ubiquitin 65.

The lumenal chaperone calreticulin contains a LIR motif 82. However, it is unclear if this participates in ER‐phagy. Calreticulin might need to be cytosolic in order to bind LC3/GABARAP and, in this event, it is uncertain how it could target the ER. Finally, overexpression of an ER‐targeted form of the mitophagy receptor Bnip3 may drive LIR‐dependent ER‐phagy, but it is not known whether this occurs endogenously 83.

## Unanswered questions in ER‐phagy

As highlighted above, several aspects of ER‐phagy mechanism and function are not yet resolved. Additional important questions are discussed further below. Addressing these areas will be important for the progress of the field. Such is the open nature of this field, and the diversity and abundance of potential viewpoints and questions arising, that the interested reader is also referred to several recent opinion articles 84, 85.

### Molecular mechanisms of ER‐phagy

#### Selectivity and receptors

The known repertoire of ER‐phagy receptors is likely incomplete and requires further elucidation, as suggested by the apparent tissue‐restricted effects of *Fam134b*,* Rtn3* and *Ccpg1* knockout 70, 76. Novel receptor(s) will have to fulfil potentially three functions, via direct activity or recruitment of other players. By definition, the receptor itself will directly mediate the recognition of the ER membrane by phagophores or lysosomes. Secondly, an ER lumenal facing or membrane‐embedded activity will function in imparting specificity for subregions of ER, or subER content. Thirdly, in the case of macroER‐phagy, deformation and scissioning of the ER membrane must be localized at site of autophagosome biogenesis; this may be coordinated by receptors. It remains to be determined how frequently these activities are encoded by separate polypeptides. While gain‐of‐function experiments, such as overexpression, have shown that receptors such as FAM134B, RTN3L and CCPG1 can drive ER‐phagy, this does not mean that all of the activities in this list are directly supplied by that molecule. For example, RHD‐containing proteins such as FAM134B and RTN3L do not span the membrane, but have the intrinsic ability to curve the ER membrane. In the case of FAM134B, cargo selection can occur via interaction of the RHD with the membrane‐embedded region of the ER chaperone calnexin. This observation also underscores that membrane‐embedded regions of receptors are not simply anchors but can also participate in scaffolding the multimolecular complexes required for ER‐phagy. Contrastingly, CCPG1 is a transmembrane protein with both an extensive lumenal domain and a cytosolic domain, unique thus far among known cargo receptors. Unlike FAM134B and RTN3L therefore, CCPG1 could potentially recognize ER content via lumenal interactions, while simultaneously linking to the cytosolic autophagy apparatus. Similar to CCPG1, TEX264 has no intrinsic membrane‐reshaping activity 78, 79. TEX264 does have a selective effect on turnover of different ER protein species 79. It is unclear whether this is mediated via interaction of TEX264, directly or indirectly, with such proteins, or whether TEX264 is restricted to activity at particular ER subregions enriched in these proteins.

Although RHD proteins such as FAM134B and RTN3L have membrane‐reshaping abilities, other receptors may not have such intrinsic activity. For instance, do CCPG1 and TEX264 have to interact directly or indirectly with ER membrane reshaping proteins in order to drive ER‐phagy? If so, would they cluster dependent upon LC3/GABARAP (and, for CCPG1, FIP200) interactions in order to fragment the ER? This is likely the case with FAM134B and RTN3L, where overexpression‐mediated fragmentation is strictly dependent upon LC3/GABARAP‐mediated clustering via intact LIR motifs 34. Even proteins with intrinsic reshaping ability, such as FAM134B and RTN3L, may interact with other RHD‐family proteins 34or, in the case of FAM134B, other reshaping proteins such as ATL2 61. Heterotypic interactions of ER‐phagy receptors with other receptors could also be necessary for optimal ER‐phagy. It might be hypothesized that coincident activation or localization of co‐operating species of receptors and/or reshaping proteins at particular ER subdomains would impart a layer of regulation on engagement of ER‐phagy in response to specific stresses. In this regard, FAM134B and TEX264 were shown to target to the same autophagosomes in response to nutrient stress; however, preliminary evidence suggests the ER‐phagy mediated by either receptor may be at least partially independent of the other 79. Uncovering determinants of clustering of membrane‐embedded receptors and ancillary proteins at autophagy initiation sites may also give deeper mechanistic insight into the triggers of ER‐phagy (and thus also cellular functions of ER‐phagy). In addition to interaction with ATG proteins, such factors could include formation of complexes with lumenal species such as unfolded proteins, or sensitivity to local ER membrane phospholipid composition or shape, lumenal redox potential or disturbances in ER lumenal flow .

For those homotypic interactors among the macroER‐phagy receptors, particularly those that have multiple ATG protein interaction sites, such as RTN3L or CCPG1, initial ATG protein‐mediated recruitment and microclustering might also promote feedforward engagement of the autophagy machinery. This would putatively occur via receptor‐mediated recruitment of further receptor and ATG proteins. For RTN3L, homotypic interaction also enhances membrane fragmentation. Furthermore, CCPG1 is a special case wherein the receptor binds two distinct proteins in the ATG hierarchy, LC3/GABARAP and FIP200. This marks out CCPG1 as unusual amongst mammalian receptors. Could this binding of ATG proteins in addition to LC3/GABARAP, FIP200 or otherwise, be a mode of action of other mammalian ER‐phagy receptors? Indeed, in this respect, two additional LC3/GABARAP‐binding autophagy receptors in mammals, NDP52 and p62/SQSTM1, were recently discovered to interact with the C‐terminus of FIP200. Recognition of mitochondria or bacteria by NDP52 results in FIP200 recruitment. In this instance, recruitment of the ULK complex via FIP200 stimulates local macroautophagy activity 87, 88. p62/SQSTM1 in protein aggregates binds FIP200 via a polypeptide sequence overlapping the LIR motif, resulting in sequential, mutually exclusive binding of FIP200 then LC3/GABARAP. This imparts directionality on the clearance of ubiqutinated protein cargo 89. It is conceivable that the dual FIP200 and LC3/GABARAP binding of CCPG1 might be involved in similar mechanism(s) in ER‐phagy.

Finally, outwith macroER‐phagy, for example, in microER‐phagy–mediated clearance of PC or the ERLAD process for ATZ clearance, molecular factors that drive budding at particular sites, and subsequent lysosomal fusion or engulfment, await complete identification. The role that ER‐lysosome contact site proteins might have in facilitating ERES‐localized microautophagy should also be considered.

#### Sites of ER‐phagy initiation

There may exist ‘hotspots’ with high potential for ER‐phagy initiation within the ER network, based upon known propensities for involvement in macroautophagy, for example, MAMs (mitochondria‐associated membranes, ER‐mitochondrion contact sites) 91 or ERESs 92. Indeed, Rab‐family GTPases, such as Ypt1/Rab1, which mediate ERES‐dependent anterograde transport from the ER, are known to play a role in yeast macroER‐phagy 93. Identifying such molecularly distinct hotspots might give further insight into the mechanisms and functions of ER‐phagy. Consideration of localized cytoskeletal dynamics in ER‐phagy may also be important. The ER is shaped by the microtubule cytoskeleton, and the role of this in ER‐phagy remains to be investigated. Yeast Lnp1 promotes ER‐phagy via the actin‐dependent encounter of Atg40 with the core Atg machinery 94. In mammalian systems, CCPG1 may have a role in regulating the RHO and CDC42 GTPases, which are master determinants of actin dynamics 74.

#### Signalling in ER‐phagy initiation

Signalling regulation of ER‐phagy also requires deeper exploration. While it is known that the canonical UPR transcriptionally regulates *CCPG1*, it is likely that other events are also involved in co‐ordination of ER‐phagy with cellular responses in different settings. Discovery of these may also give further insight into the cellular functions of ER‐phagy. For example, are ER‐phagy receptors or other ER membrane proteins post‐translationally modified? This is a highly attractive option given that the cytosolic surface of the ER acts as a scaffold for cell signalling pathways. Ubiquitination is a prime candidate, as ubiquitin‐dependent and ‐independent modes of selective autophagy have been described within the pantheon of other selective ER‐phagy pathways 95. This diversity might also exist between different forms of ER‐phagy. Phosphorylation and acetylation of cargo or receptors is also prevalent in other selective autophagy paradigms but remains to be addressed for ER‐phagy. For example, the binding affinity of Optineurin for LC3/GABARAP and polyubiquitinated cargo is modulated via phosphorylation near the core LIR motif and in the UBAN (Ub‐binding domain in ABIN proteins and NEMO) domain, respectively 40. Finally, are other ER status‐sensing relays implicated in ER‐phagy? Examples of the latter that might be tested include non‐canonical UPR‐driven gene sets activated by lipid bilayer abnormalities , or Ca^2+^ and NF‐κB (Nuclear Factor‐κB)‐driven signalling resulting from ER protein overload 110.

### Functions of ER‐phagy

#### Proteostasis

Investigations of the cellular functions of ER‐phagy have uncovered roles in proteostasis and UPR regulation, as outlined above, including specific targeting of PC by ER‐phagy and ATZ by ER‐phagy related ERLAD. Furthermore, mutant gonadotrophin‐releasing hormone receptor (GnRHR) is degraded by autophagy and thus potentially by ER‐phagy 111. However, mutant GnRHR may be incorporated from the ER membrane into the delimiting membrane of autophagosomes in a form of ERLAD for which mechanistic details are unclear. Similarly, another ERAD‐resistant aberrant lumenal protein species for which there is evidence of lysosomal degradation, but where the role of macroER‐phagy is unclear, is the lumenal granule of beta subunits of thyrotrophic hormone in the secretory cells of the stimulated pituitary gland. In fact, the existing morphological evidence suggests a similar pathway to the ER‐phagy–related ERLAD process that removes ATZ 112. Mutant dysferlin in muscle cells is another potential target of ERLAD 113. In another example of putative proteostatic roles, antibody‐secreting plasma cells require core ATG proteins to manage immunoglobulin synthesis 55. In the absence of ATG proteins, an expanded ER is observed, concomitant with excess immunoglobulin synthesis and secretion. *Ccpg1* is highly upregulated during the differentiation of these cells, suggesting a potential role for proteostatic ER‐phagy 114. Indeed, this would represent an important role for ER‐phagy in health, given the critical role of immunoglobulin secretion in immune surveillance. Potentially, ER‐phagy might be optimal at a ‘sweet spot’ level where ER volume and immunoglobulin secretion would be at the maximal level tolerated without cellular toxicity. This is an area of ER‐phagy biology that warrants urgent investigation.

#### Other potential roles including innate immunity

Notwithstanding its clear involvement in proteostasis, other potential roles for ER‐phagy should be addressed. For example, does ER‐phagy remodel the ER in order to: determine the capacity for steroid hormone or phospholipid synthesis; resolve topological perturbations of the network; regulate calcium homeostasis; regulate platforming of cellular signalling or regulate organelle contact site‐dependent processes? ER‐phagy is induced by lipotoxic stress in cultured HepG2 hepatocytes but its relevance is unclear 114. Perhaps most strikingly, macroER‐phagy has been implicated in generating ‘signalling’ phagophores that scaffold activation of TBK1 (TANK‐binding kinase 1) by its upstream regulator stimulator of interferon genes (STING) in order to co‐ordinate the cellular response to bacterial infection 114. It appears that molecular patterns associated with some live bacteria are detected by STING, resulting in UPR signalling and induction of ER‐phagy. The generation of autophagy structures containing a variety of ER components and STING seems to be required for TBK1 activation and interferon responses. It is presumed that early, unsealed autophagosomes provide a signalling scaffold for STING‐TBK1 signalling out into the cytosol. Indeed, regardless of the former mechanism, a wider ER‐phagy involvement in innate immune responses to infection is currently emerging, in addition to the aforementioned potential role of ER‐phagy during immunoglobulin production in adaptive immunity. For example, FAM134B suppresses proliferation of Ebolavirus in MEFs 114, and Flaviviruses in human brain microvascular endothelial cells 114. The Flavivirus protease NS2B3 mediates cleavage of FAM134B to prevent virion sequestration in ER‐derived autophagosomes. *Ccpg1* is induced in intestinal Paneth cells in response to Norovirus infection, also suggestive of a role in host defence 114. Herpes simplex virus type I (HSV‐1)‐infected macrophages may sequester virions into nuclear membrane‐derived vesicles that incorporate pieces of nuclear membrane within. However, it is unclear if the efficiency of membrane fragment sequestration is sufficient to class this as selective ER‐phagy 114. Interestingly, micronuclei are degraded by macroautophagy, suggesting that the ER‐derived membranes around these organelles may be involved in this process 114.

#### ER‐phagy in cancer and ageing

Cancers should be assessed for changes in ER‐phagy molecule expression and function. Already, mutations and alterations in FAM134B expression have been observed in various malignancies 114. Functionally, FAM134B loss may promote colorectal cancer cell tumourigenicity 114. Conversely, glioma cells bearing *IDH1* mutations may require FAM134B‐driven ER‐phagy to survive proteotoxic stress, framing this as a synthetic lethal therapeutic target 114. The SEC62 gene is amplified in a number of cancers, including lung adenocarcinomas, prostate, thyroid and head and neck squamous cell carcinoma; accordingly it has been hypothesized that excess SEC62 may not be incorporated into SEC61 complexes and instead lead to sensitized ER‐phagy responses and, consequently, resistance to anticancer ER stress 114. Finally, defective ER‐phagy may also be involved in ageing, as suggested by a mouse model of progeria driven by *Slc33a1* overexpression, but further investigation is required 114.

To finish, a caveat should be noted in regard of the above questions and observations; once an ER‐phagy protein is implicated in a given phenomenon or disease, it is important to mechanistically ascertain whether this is due to defective ER‐phagy or whether the protein serves to regulate the ER via other, co‐ordinated functions. This requires more sophisticated experimentation than simple gene knockout. Nonetheless, overall, ER‐phagy is clearly of importance in health and disease, and its roles will be clarified by future studies.

## Conclusions

Identification of molecularly distinct pathways for ER degradation by ER‐phagy and related processes has allowed elucidation of key principles of mechanism. As well as cargo receptors, a rich mix of other players participates, including chaperones and membrane reshaping molecules. Distinct ER‐phagy pathways play diverse roles in different cell types and are implicated in disease aetiologies. Excitingly, the field has only just begun to uncover the full complement of ER‐phagy mechanisms and functions.

## Conflict of interest

The authors declare no conflict of interest.

## References

[1] English AR , Zurek N & Voeltz GK (2009) Peripheral ER structure and function. Curr Opin Cell Biol 21, 596–602. 19447593 10.1016/j.ceb.2009.04.004 PMC2753178

[2] Phillips MJ & Voeltz GK (2016) Structure and function of ER membrane contact sites with other organelles. Nat Rev Mol Cell Biol 17, 69–82. 26627931 10.1038/nrm.2015.8 PMC5117888

[3] Walter P & Ron D (2011) The unfolded protein response: from stress pathway to homeostatic regulation. Science 334, 1081–1086. 22116877 10.1126/science.1209038

[4] Ruggiano A , Foresti O & Carvalho P (2014) Quality control: ER‐associated degradation: protein quality control and beyond. J Cell Biol 204, 869–879. 24637321 10.1083/jcb.201312042 PMC3998802

[5] Caldieri G , Barbieri E , Nappo G , Raimondi A , Bonora M , Conte A , Verhoef L , Confalonieri S , Malabarba MG , Bianchi F , et al. (2017) Reticulon 3-dependent ER-PM contact sites control EGFR nonclathrin endocytosis. Science 356, 617–624. 28495747 10.1126/science.aah6152 PMC5432029

[6] Cubillos‐Ruiz JR , Bettigole SE & Glimcher LH (2017) Tumorigenic and immunosuppressive effects of endoplasmic reticulum stress in cancer. Cell 168, 692–706. 28187289 10.1016/j.cell.2016.12.004 PMC5333759

[7] Hotamisligil GS (2010) Endoplasmic reticulum stress and the inflammatory basis of metabolic disease. Cell 140, 900–917. 20303879 10.1016/j.cell.2010.02.034 PMC2887297

[8] Wang M & Kaufman RJ (2016) Protein misfolding in the endoplasmic reticulum as a conduit to human disease. Nature 529, 326–335. 26791723 10.1038/nature17041

[9] Todd DJ , McHeyzer-Williams LJ , Kowal C , Lee AH , Volpe BT , Diamond B , McHeyzer-Williams MG & Glimcher LH (2009) XBP1 governs late events in plasma cell differentiation and is not required for antigen‐specific memory B cell development. J Exp Med 206, 2151 – 2159. 19752183 10.1084/jem.20090738 PMC2757870

[10] Huh WJ , Esen E , Geahlen JH , Bredemeyer AJ , Lee AH , Shi G , Konieczny SF , Glimcher LH & Mills JC (2010) XBP1 controls maturation of gastric zymogenic cells by induction of MIST1 and expansion of the rough endoplasmic reticulum. Gastroenterology 139, 2038 – 2049. 20816838 10.1053/j.gastro.2010.08.050 PMC2997137

[11] Smith M & Wilkinson S (2017) ER homeostasis and autophagy. Essays Biochem 61, 625–635. 29233873 10.1042/EBC20170092 PMC5869861

[12] Oku M & Sakai Y (2018) Three distinct types of microautophagy based on membrane dynamics and molecular machineries. BioEssays 40, e1800008. 29708272 10.1002/bies.201800008

[13] Dikic I & Elazar Z (2018) Mechanism and medical implications of mammalian autophagy. Nat Rev Mol Cell Biol 19, 349–364. 29618831 10.1038/s41580-018-0003-4

[14] Ogata M , Hino S , Saito A , Morikawa K , Kondo S , Kanemoto S , Murakami T , Taniguchi M , Tanii I , Yoshinaga K , et al. (2006) Autophagy is activated for cell survival after endoplasmic reticulum stress. Mol Cell Biol 26, 9220–9231. 17030611 10.1128/MCB.01453-06 PMC1698520

[15] Nakatogawa H & Mochida K (2015) Reticulophagy and nucleophagy: new findings and unsolved issues. Autophagy 11, 2377–2378. 26566146 10.1080/15548627.2015.1106665 PMC4835146

[16] Locke M & Collins JV (1965) The structure and formation of protein granules in the fat body of an insect. J Cell Biol 26, 857–884. 19866685 10.1083/jcb.26.3.857 PMC2106788

[17] Liu Y , Burgos JS , Deng Y , Srivastava R , Howell SH & Bassham DC (2012) Degradation of the endoplasmic reticulum by autophagy during endoplasmic reticulum stress in *Arabidopsis* . Plant Cell 24, 4635 – 4651. 23175745 10.1105/tpc.112.101535 PMC3531857

[18] Anding AL & Baehrecke EH (2017) Cleaning house: selective autophagy of organelles. Dev Cell 41, 10–22. 28399394 10.1016/j.devcel.2017.02.016 PMC5395098

[19] Lamb CA , Yoshimori T & Tooze SA (2013) The autophagosome: origins unknown, biogenesis complex. Nat Rev Mol Cell Biol 14, 759–774. 24201109 10.1038/nrm3696

[20] Ganley IG , Lam du H , Wang J , Ding X , Chen S & Jiang X (2009) ULK1.ATG13.FIP200 complex mediates mTOR signaling and is essential for autophagy. J Biol Chem 284, 12297 – 12305. 19258318 10.1074/jbc.M900573200 PMC2673298

[21] Zachari M & Ganley IG (2017) The mammalian ULK1 complex and autophagy initiation. Essays Biochem 61, 585–596. 29233870 10.1042/EBC20170021 PMC5869855

[22] Hayashi-Nishino M , Fujita N , Noda T , Yamaguchi A , Yoshimori T & Yamamoto A (2009) A subdomain of the endoplasmic reticulum forms a cradle for autophagosome formation. Nat Cell Biol 11, 1433 – 1437. 19898463 10.1038/ncb1991

[23] Yla-Anttila P , Vihinen H , Jokitalo E & Eskelinen EL (2009) 3D tomography reveals connections between the phagophore and endoplasmic reticulum. Autophagy 5, 1180–1185. 19855179 10.4161/auto.5.8.10274

[24] Polson HEJ , de Lartigue J , Rigden DJ , Reedijk M , Urbe S , Clague MJ & Tooze SA (2010) Mammalian Atg18 (WIPI2) localizes to omegasome-anchored phagophores and positively regulates LC3 lipidation. Autophagy 6, 506–522. 20505359 10.4161/auto.6.4.11863

[25] Papinski D , Schuschnig M , Reiter W , Wilhelm L , Barnes CA , Maiolica A , Hansmann I , Pfaffenwimmer T , Kijanska M , Stoffel I , et al. (2014) Early steps in autophagy depend on direct phosphorylation of Atg9 by the Atg1 kinase. Mol Cell 53, 471–483. 24440502 10.1016/j.molcel.2013.12.011 PMC3978657

[26] Puri C , Renna M , Bento CF , Moreau K & Rubinsztein DC (2013) Diverse autophagosome membrane sources coalesce in recycling endosomes. Cell 154, 1285–1299. 24034251 10.1016/j.cell.2013.08.044 PMC3791395

[27] Yamamoto H , Kakuta S , Watanabe TM , Kitamura A , Sekito T , Kondo-Kakuta C , Ichikawa R , Kinjo M & Ohsumi Y (2012) Atg9 vesicles are an important membrane source during early steps of autophagosome formation. J Cell Biol 198, 219–233. 22826123 10.1083/jcb.201202061 PMC3410421

[28] Dooley HC , Razi M , Polson HEJ , Girardin SE , Wilson MI & Tooze SA (2014) WIPI2 links LC3 conjugation with PI3P, autophagosome formation, and pathogen clearance by recruiting Atg12-5-16L1. Mol Cell 55, 238–252. 24954904 10.1016/j.molcel.2014.05.021 PMC4104028

[29] Gammoh N , Florey O , Overholtzer M & Jiang X (2013) Interaction between FIP200 and ATG16L1 distinguishes ULK1 complex-dependent and -independent autophagy. Nat Struct Mol Biol 20, 144–149. 23262492 10.1038/nsmb.2475 PMC3565010

[30] Codogno P , Mehrpour M & Proikas‐Cezanne T (2011) Canonical and non‐canonical autophagy: variations on a common theme of self‐eating? Nat Rev Mol Cell Biol 13, 7–12. 22166994 10.1038/nrm3249

[31] Martinez J , Almendinger J , Oberst A , Ness R , Dillon CP , Fitzgerald P , Hengartner MO & Green DR (2011) Microtubule-associated protein 1 light chain 3 alpha (LC3)-associated phagocytosis is required for the efficient clearance of dead cells. Proc Natl Acad Sci USA 108, 17396–17401. 21969579 10.1073/pnas.1113421108 PMC3198353

[32] Sanjuan MA , Dillon CP , Tait SW , Moshiach S , Dorsey F , Connell S , Komatsu M , Tanaka K , Cleveland JL , Withoff S , et al. (2007) Toll-like receptor signalling in macrophages links the autophagy pathway to phagocytosis. Nature 450, 1253–1257. 18097414 10.1038/nature06421

[33] Khaminets A , Heinrich T , Mari M , Grumati P , Huebner AK , Akutsu M , Liebmann L , Stolz A , Nietzsche S , Koch N , et al. (2015) Regulation of endoplasmic reticulum turnover by selective autophagy. Nature 522, 354–358. 26040720 10.1038/nature14498

[34] Grumati P , Morozzi G , Holper S , Mari M , Harwardt MI , Yan R , Muller S , Reggiori F , Heilemann M & Dikic I (2017) Full length RTN3 regulates turnover of tubular endoplasmic reticulum via selective autophagy. Elife 6, e25555. 28617241 10.7554/eLife.25555 PMC5517149

[35] Rogov V , Dotsch V , Johansen T & Kirkin V (2014) Interactions between autophagy receptors and ubiquitin-like proteins form the molecular basis for selective autophagy. Mol Cell 53, 167–178. 24462201 10.1016/j.molcel.2013.12.014

[36] Birgisdottir AB , Lamark T & Johansen T (2013) The LIR motif – crucial for selective autophagy. J Cell Sci 126 (Pt 15), 3237–3247. 23908376 10.1242/jcs.126128

[37] Rogov VV , Stolz A , Ravicahandran AC , Rios-Szwed DO , Suzuki H , Kniss A , Lohr F , Wakatsuki S , Dotsch V , Dikic I , et al. (2017) Structural and functional analysis of the GABARAP interaction motif (GIM). EMBO Rep 18, 1382–1396. 28655748 10.15252/embr.201643587 PMC5538626

[38] Farre JC & Subramani S (2016) Mechanistic insights into selective autophagy pathways: lessons from yeast. Nat Rev Mol Cell Biol 17, 537–552. 27381245 10.1038/nrm.2016.74 PMC5549613

[39] Richter B , Sliter DA , Herhaus L , Stolz A , Wang C , Beli P , Zaffagnini G , Wild P , Martens S , Wagner SA , et al. (2016) Phosphorylation of OPTN by TBK1 enhances its binding to Ub chains and promotes selective autophagy of damaged mitochondria. Proc Natl Acad Sci USA 113, 4039–4044. 27035970 10.1073/pnas.1523926113 PMC4839414

[40] Wild P , Farhan H , McEwan DG , Wagner S , Rogov VV , Brady NR , Richter B , Korac J , Waidmann O , Choudhary C , et al. (2011) Phosphorylation of the autophagy receptor optineurin restricts Salmonella growth. Science 333, 228–233. 21617041 10.1126/science.1205405 PMC3714538

[41] Mijaljica D , Prescott M & Devenish RJ (2011) Microautophagy in mammalian cells: revisiting a 40‐year‐old conundrum. Autophagy 7, 673–682. 21646866 10.4161/auto.7.7.14733

[42] Guan J , Stromhaug PE , George MD , Habibzadegah-Tari P , Bevan A , Dunn WA Jr & Klionsky DJ (2001) Cvt18/Gsa12 is required for cytoplasm-to-vacuole transport, pexophagy, and autophagy in *Saccharomyces cerevisiae* and *Pichia pastoris* . Mol Biol Cell 12, 3821–3838. 11739783 10.1091/mbc.12.12.3821 PMC60758

[43] Fry MR , Thomson JM , Tomasini AJ & Dunn WA Jr (2006) Early and late molecular events of glucose-induced pexophagy in *Pichia pastoris* require Vac8. Autophagy 2, 280–288. 16921262 10.4161/auto.3164

[44] Oku M , Nishimura T , Hattori T , Ano Y , Yamashita S & Sakai Y (2006) Role of Vac8 in formation of the vacuolar sequestering membrane during micropexophagy. Autophagy 2, 272–279. 16874085 10.4161/auto.3135

[45] Farre JC & Subramani S (2004) Peroxisome turnover by micropexophagy: an autophagy‐related process. Trends Cell Biol 14, 515–523. 15350980 10.1016/j.tcb.2004.07.014

[46] Farre JC , Manjithaya R , Mathewson RD & Subramani S (2008) PpAtg30 tags peroxisomes for turnover by selective autophagy. Dev Cell 14, 365–376. 18331717 10.1016/j.devcel.2007.12.011 PMC3763908

[47] Sahu R , Kaushik S , Clement CC , Cannizzo ES , Scharf B , Follenzi A , Potolicchio I , Nieves E , Cuervo AM & Santambrogio L (2011) Microautophagy of cytosolic proteins by late endosomes. Dev Cell 20, 131–139. 21238931 10.1016/j.devcel.2010.12.003 PMC3025279

[48] Oku M , Maeda Y , Kagohashi Y , Kondo T , Yamada M , Fujimoto T & Sakai Y (2017) Evidence for ESCRT- and clathrin-dependent microautophagy. J Cell Biol 216, 3263–3274. 28838958 10.1083/jcb.201611029 PMC5626533

[49] Liu XM , Sun LL , Hu W , Ding YH , Dong MQ & Du LL (2015) ESCRTs cooperate with a selective autophagy receptor to mediate vacuolar targeting of soluble cargos. Mol Cell 59, 1035–1042. 26365378 10.1016/j.molcel.2015.07.034

[50] Mejlvang J , Olsvik H , Svenning S , Bruun JA , Abudu YP , Larsen KB , Brech A , Hansen TE , Brenne H , Hansen T , et al. (2018) Starvation induces rapid degradation of selective autophagy receptors by endosomal microautophagy. J Cell Biol 217, 3640–3655. 30018090 10.1083/jcb.201711002 PMC6168274

[51] Bolender RP & Weibel ER (1973) A morphometric study of the removal of phenobarbital‐induced membranes from hepatocytes after cessation of threatment. J Cell Biol 56, 746–761. 4569312 10.1083/jcb.56.3.746 PMC2108940

[52] Tooze J , Hollinshead M , Ludwig T , Howell K , Hoflack B & Kern H (1990) In exocrine pancreas, the basolateral endocytic pathway converges with the autophagic pathway immediately after the early endosome. J Cell Biol 111, 329–345. 2166050 10.1083/jcb.111.2.329 PMC2116176

[53] Bernales S , McDonald KL & Walter P (2006) Autophagy counterbalances endoplasmic reticulum expansion during the unfolded protein response. PLoS Biol 4, e423. 17132049 10.1371/journal.pbio.0040423 PMC1661684

[54] Schuck S , Gallagher CM & Walter P (2014) ER‐phagy mediates selective degradation of endoplasmic reticulum independently of the core autophagy machinery. J Cell Sci 127 (Pt 18), 4078–4088. 25052096 10.1242/jcs.154716 PMC4163648

[55] Pengo N , Scolari M , Oliva L , Milan E , Mainoldi F , Raimondi A , Fagioli C , Merlini A , Mariani E , Pasqualetto E , et al. (2013) Plasma cells require autophagy for sustainable immunoglobulin production. Nat Immunol 14, 298–305. 23354484 10.1038/ni.2524

[56] Antonucci L , Fagman JB , Kim JY , Todoric J , Gukovsky I , Mackey M , Ellisman MH & Karin M (2015) Basal autophagy maintains pancreatic acinar cell homeostasis and protein synthesis and prevents ER stress. Proc Natl Acad Sci USA 112, E6166–E6174. 26512112 10.1073/pnas.1519384112 PMC4653219

[57] Diakopoulos KN , Lesina M , Wormann S , Song L , Aichler M , Schild L , Artati A , Romisch-Margl W , Wartmann T , Fischer R , et al. (2015) Impaired autophagy induces chronic atrophic pancreatitis in mice via sex- and nutrition-dependent processes. Gastroenterology 148, 626–638.e17. 25497209 10.1053/j.gastro.2014.12.003

[58] Jia W , Pua HH , Li QJ & He YW (2011) Autophagy regulates endoplasmic reticulum homeostasis and calcium mobilization in T lymphocytes. J Immunol 186, 1564–1574. 21191072 10.4049/jimmunol.1001822 PMC3285458

[59] Cinque L , Forrester A , Bartolomeo R , Svelto M , Venditti R , Montefusco S , Polishchuk E , Nusco E , Rossi A , Medina DL , et al. (2010) The heterogeneity of cell subtypes from a primary culture of human amniotic fluid. Cell.

[60] Zhang H & Hu J (2016) Shaping the endoplasmic reticulum into a social network. Trends Cell Biol 26, 934–943. 27339937 10.1016/j.tcb.2016.06.002

[61] Liang JR , Lingeman E , Ahmed S & Corn JE (2018) Atlastins remodel the endoplasmic reticulum for selective autophagy. J Cell Biol 217, 3354–3367. 30143524 10.1083/jcb.201804185 PMC6168278

[62] Forrester A , De Leonibus C , Grumati P , Fasana E , Piemontese M , Staiano L , Fregno I , Raimondi A , Marazza A , Bruno G , et al. (2019) A selective ER-phagy exerts procollagen quality control via a Calnexin-FAM134B complex. EMBO J 38, e99847. 30559329 10.15252/embj.201899847 PMC6331724

[63] Fregno I , Fasana E , Bergmann TJ , Raimondi A , Loi M , Solda T , Galli C , D'Antuono R , Morone D , Danieli A , et al. (2018) ER-to-lysosome-associated degradation of proteasome-resistant ATZ polymers occurs via receptor-mediated vesicular transport. EMBO J 37, e99259. 30076131 10.15252/embj.201899259 PMC6120659

[64] Schultz ML , Krus KL , Kaushik S , Dang D , Chopra R , Qi L , Shakkottai VG , Cuervo AM & Lieberman AP (2018) Coordinate regulation of mutant NPC1 degradation by selective ER autophagy and MARCH6-dependent ERAD. Nat Commun 9, 3671. 30202070 10.1038/s41467-018-06115-2 PMC6131187

[65] Omari S , Makareeva E , Roberts-Pilgrim A , Mirigian L , Jarnik M , Ott C , Lippincott-Schwartz J & Leikin S (2018) Noncanonical autophagy at ER exit sites regulates procollagen turnover. Proc Natl Acad Sci USA 115, E10099–E10108. 30287488 10.1073/pnas.1814552115 PMC6205486

[66] Mochida K , Oikawa Y , Kimura Y , Kirisako H , Hirano H , Ohsumi Y & Nakatogawa H (2015) Receptor-mediated selective autophagy degrades the endoplasmic reticulum and the nucleus. Nature 522, 359–362. 26040717 10.1038/nature14506

[67] Kurth I , Pamminger T , Hennings JC , Soehendra D , Huebner AK , Rotthier A , Baets J , Senderek J , Topaloglu H , Farrell SA , et al. (2009) Mutations in FAM134B, encoding a newly identified Golgi protein, cause severe sensory and autonomic neuropathy. Nat Genet 41, 1179–1181. 19838196 10.1038/ng.464

[68] Voeltz GK , Prinz WA , Shibata Y , Rist JM & Rapoport TA (2006) A class of membrane proteins shaping the tubular endoplasmic reticulum. Cell 124, 573–586. 16469703 10.1016/j.cell.2005.11.047

[69] Schroeder LK , Barentine AES , Merta H , Schweighofer S , Zhang Y , Baddeley D , Bewersdorf J & Bahmanyar S (2019) Dynamic nanoscale morphology of the ER surveyed by STED microscopy. J Cell Biol 218, 83–96. 30442642 10.1083/jcb.201809107 PMC6314542

[70] Shi Q , Ge Y , Sharoar MG , He W , Xiang R , Zhang Z , Hu X & Yan R (2014) Impact of RTN3 deficiency on expression of BACE1 and amyloid deposition. J Neurosci 34, 13954–13962. 25319692 10.1523/JNEUROSCI.1588-14.2014 PMC4198539

[71] Chen Q , Xiao Y , Chai P , Zheng P , Teng J & Chen J (2019) ATL3 is a tubular ER-phagy receptor for GABARAP-mediated selective autophagy. Curr Biol 29, 846–855.e6. 30773365 10.1016/j.cub.2019.01.041

[72] Durr A , Camuzat A , Colin E , Tallaksen C , Hannequin D , Coutinho P , Fontaine B , Rossi A , Gil R , Rousselle C , et al. (2004) Atlastin1 mutations are frequent in young-onset autosomal dominant spastic paraplegia. Arch Neurol 61, 1867–1872. 15596607 10.1001/archneur.61.12.1867

[73] Fumagalli F , Noack J , Bergmann TJ , Cebollero E , Pisoni GB , Fasana E , Fregno I , Galli C , Loi M , Solda T , et al. (2016) Translocon component Sec62 acts in endoplasmic reticulum turnover during stress recovery. Nat Cell Biol 18, 1173–1184. 27749824 10.1038/ncb3423

[74] Kostenko EV , Olabisi OO , Sahay S , Rodriguez PL & Whitehead IP (2006) Ccpg1, a novel scaffold protein that regulates the activity of the Rho guanine nucleotide exchange factor Dbs. Mol Cell Biol 26, 8964–8975. 17000758 10.1128/MCB.00670-06 PMC1636807

[75] Smith MD & Wilkinson S (2018) CCPG1, a cargo receptor required for reticulophagy and endoplasmic reticulum proteostasis. Autophagy 14, 1090–1091. 29916296 10.1080/15548627.2018.1441473 PMC6103402

[76] Smith MD , Harley ME , Kemp AJ , Wills J , Lee M , Arends M , von Kriegsheim A , Behrends C & Wilkinson S (2018) CCPG1 is a non-canonical autophagy cargo receptor essential for ER-phagy and pancreatic ER proteostasis. Dev Cell 44, 217–232.e11. 29290589 10.1016/j.devcel.2017.11.024 PMC5791736

[77] Grumati P , Dikic I & Stolz A (2018) ER‐phagy at a glance. J Cell Sci 131, jcs217364. 30177506 10.1242/jcs.217364

[78] Chino H , Hatta T , Natsume T & Mizushima N (2019) Intrinsically disordered protein TEX264 mediates ER-phagy. Mol Cell. 10.1016/j.molcel.2019.03.033 31006538

[79] An H , Ordureau A , Paulo JA , Shoemaker CJ , Denic V & Harper JW (2019) TEX264 is an endoplasmic reticulum-resident ATG8-interacting protein critical for ER remodeling during nutrient stress. Mol Cell. 10.1016/j.molcel.2019.03.034 PMC6747008 31006537

[80] Yang H , Ni HM , Guo F , Ding Y , Shi YH , Lahiri P , Frohlich LF , Rulicke T , Smole C , Schmidt VC , et al. (2016) Sequestosome 1/p62 protein is associated with autophagic removal of excess hepatic endoplasmic reticulum in mice. J Biol Chem 291, 18663–18674. 27325701 10.1074/jbc.M116.739821 PMC5009243

[81] Tschurtschenthaler M , Adolph TE , Ashcroft JW , Niederreiter L , Bharti R , Saveljeva S , Bhattacharyya J , Flak MB , Shih DQ , Fuhler GM , et al. (2017) Defective ATG16L1-mediated removal of IRE1alpha drives Crohn's disease-like ileitis. J Exp Med 214, 401–422. 28082357 10.1084/jem.20160791 PMC5294857

[82] Yang Y , Ma F , Liu Z , Su Q , Liu Y , Liu Z & Li Y (2019) The ER-localized Ca2+-binding protein calreticulin couples ER stress to autophagy by associating with microtubule-associated protein 1A/1B light chain 3. J Biol Chem 294, 772–782. 30429217 10.1074/jbc.RA118.005166 PMC6341397

[83] Hanna RA , Quinsay MN , Orogo AM , Giang K , Rikka S & Gustafsson AB (2012) Microtubule-associated protein 1 light chain 3 (LC3) interacts with Bnip3 protein to selectively remove endoplasmic reticulum and mitochondria via autophagy. J Biol Chem 287, 19094–19104. 22505714 10.1074/jbc.M111.322933 PMC3365942

[84] Fregno I & Molinari M (2018) Endoplasmic reticulum turnover: ER‐phagy and other flavors in selective and non‐selective ER clearance. F1000Res 7, 454. 29744037 10.12688/f1000research.13968.1 PMC5904726

[85] Dikic I (2018) Open questions: why should we care about ER‐phagy and ER remodelling? BMC Biol 16, 131. 30382915 10.1186/s12915-018-0603-7 PMC6211458

[86] Holcman D , Parutto P , Chambers JE , Fantham M , Young LJ , Marciniak SJ , Kaminski CF , Ron D & Avezov E (2018) Single particle trajectories reveal active endoplasmic reticulum luminal flow. Nat Cell Biol 20, 1118–1125. 30224760 10.1038/s41556-018-0192-2 PMC6435195

[87] Ravenhill BJ , Boyle KB , von Muhlinen N , Ellison CJ , Masson GR , Otten EG , Foeglein A , Williams R & Randow F (2019) The cargo receptor NDP52 initiates selective autophagy by recruiting the ULK complex to cytosol-invading bacteria. Mol Cell 74, 320–329.e6. 30853402 10.1016/j.molcel.2019.01.041 PMC6477152

[88] Vargas JNS , Wang C , Bunker E , Hao L , Maric D , Schiavo G , Randow F & Youle RJ (2019) Spatiotemporal control of ULK1 activation by NDP52 and TBK1 during selective autophagy. Mol Cell 74, 347–362.e6. 30853401 10.1016/j.molcel.2019.02.010 PMC6642318

[89] Turco E , Witt M , Abert C , Bock-Bierbaum T , Su MY , Trapannone R , Sztacho M , Danieli A , Shi X , Zaffagnini G , et al. (2019) FIP200 claw domain binding to p62 promotes autophagosome formation at ubiquitin condensates. Mol Cell 74, 330–346.e11. 30853400 10.1016/j.molcel.2019.01.035 PMC6477179

[90] Hamasaki M , Furuta N , Matsuda A , Nezu A , Yamamoto A , Fujita N , Oomori H , Noda T , Haraguchi T , Hiraoka Y , et al. (2013) Autophagosomes form at ER-mitochondria contact sites. Nature 495, 389–393. 23455425 10.1038/nature11910

[91] Hailey DW , Rambold AS , Satpute-Krishnan P , Mitra K , Sougrat R , Kim PK & Lippincott-Schwartz J (2010) Mitochondria supply membranes for autophagosome biogenesis during starvation. Cell 141, 656–667. 20478256 10.1016/j.cell.2010.04.009 PMC3059894

[92] Ge L , Zhang M , Kenny SJ , Liu D , Maeda M , Saito K , Mathur A , Xu K & Schekman R (2017) Remodeling of ER-exit sites initiates a membrane supply pathway for autophagosome biogenesis. EMBO Rep 18, 1586–1603. 28754694 10.15252/embr.201744559 PMC5579361

[93] Lipatova Z & Segev N (2015) A role for macro‐ER‐phagy in ER quality control. PLoS Genet 11, e1005390. 26181331 10.1371/journal.pgen.1005390 PMC4504476

[94] Chen S , Cui Y , Parashar S , Novick PJ & Ferro-Novick S (2018) ER-phagy requires Lnp1, a protein that stabilizes rearrangements of the ER network. Proc Natl Acad Sci USA 115, E6237–E6244. 29915089 10.1073/pnas.1805032115 PMC6142256

[95] Khaminets A , Behl C & Dikic I (2016) Ubiquitin‐dependent and independent signals in selective autophagy. Trends Cell Biol 26, 6–16. 26437584 10.1016/j.tcb.2015.08.010

[96] Koh JH , Wang L , Beaudoin-Chabot C & Thibault G (2018) Lipid bilayer stress-activated IRE-1 modulates autophagy during endoplasmic reticulum stress. J Cell Sci 131, jcs217992. 30333136 10.1242/jcs.217992

[97] Pahl HL & Baeuerle PA (1997) The ER‐overload response: activation of NF‐kappa B. Trends Biochem Sci 22, 63–67. 9048485 10.1016/s0968-0004(96)10073-6

[98] Houck SA , Ren HY , Madden VJ , Bonner JN , Conlin MP , Janovick JA , Conn PM & Cyr DM (2014) Quality control autophagy degrades soluble ERAD-resistant conformers of the misfolded membrane protein GnRHR. Mol Cell 54, 166–179. 24685158 10.1016/j.molcel.2014.02.025 PMC4070183

[99] Noda T & Farquhar MG (1992) A non‐autophagic pathway for diversion of ER secretory proteins to lysosomes. J Cell Biol 119, 85–97. 1527175 10.1083/jcb.119.1.85 PMC2289624

[100] Fujita E , Kouroku Y , Isoai A , Kumagai H , Misutani A , Matsuda C , Hayashi YK & Momoi T (2007) Two endoplasmic reticulum-associated degradation (ERAD) systems for the novel variant of the mutant dysferlin: ubiquitin/proteasome ERAD(I) and autophagy/lysosome ERAD(II). Hum Mol Genet 16, 618–629. 17331981 10.1093/hmg/ddm002

[101] Shi W , Liao Y , Willis SN , Taubenheim N , Inouye M , Tarlinton DM , Smyth GK , Hodgkin PD , Nutt SL & Corcoran LM (2015) Transcriptional profiling of mouse B cell terminal differentiation defines a signature for antibody-secreting plasma cells. Nat Immunol 16, 663–673. 25894659 10.1038/ni.3154

[102] Pang L , Liu K , Liu D , Lv F , Zang Y , Xie F , Yin J , Shi Y , Wang Y & Chen D (2018) Differential effects of reticulophagy and mitophagy on nonalcoholic fatty liver disease. Cell Death Dis 9, 90. 29367738 10.1038/s41419-017-0136-y PMC5833629

[103] Moretti J , Roy S , Bozec D , Martinez J , Chapman JR , Ueberheide B , Lamming DW , Chen ZJ , Horng T , Yeretssian G , et al. (2017) STING senses microbial viability to orchestrate stress-mediated autophagy of the endoplasmic reticulum. Cell 171, 809–823.e13. 29056340 10.1016/j.cell.2017.09.034 PMC5811766

[104] Chiramel AI , Dougherty JD , Nair V , Robertson SJ & Best SM (2016) FAM134B, the selective autophagy receptor for endoplasmic reticulum turnover, inhibits replication of Ebola virus strains Makona and Mayinga. J Infect Dis 214 (Suppl 3), S319–S325. 27511895 10.1093/infdis/jiw270 PMC5050481

[105] Lennemann NJ & Coyne CB (2017) Dengue and Zika viruses subvert reticulophagy by NS2B3‐mediated cleavage of FAM134B. Autophagy 13, 322–332. 28102736 10.1080/15548627.2016.1265192 PMC5324851

[106] Cadwell K , Patel KK , Maloney NS , Liu TC , Ng AC , Storer CE , Head RD , Xavier R , Stappenbeck TS & Virgin HW (2010) Virus-plus-susceptibility gene interaction determines Crohn's disease gene Atg16L1 phenotypes in intestine. Cell 141, 1135–1145. 20602997 10.1016/j.cell.2010.05.009 PMC2908380

[107] English L , Chemali M , Duron J , Rondeau C , Laplante A , Gingras D , Alexander D , Leib D , Norbury C , Lippe R , et al. (2009) Autophagy enhances the presentation of endogenous viral antigens on MHC class I molecules during HSV-1 infection. Nat Immunol 10, 480–487. 19305394 10.1038/ni.1720 PMC3885169

[108] Rello-Varona S , Lissa D , Shen S , Niso-Santano M , Senovilla L , Marino G , Vitale I , Jemaa M , Harper F , Pierron G , et al. (2012) Autophagic removal of micronuclei. Cell Cycle 11, 170–176. 22185757 10.4161/cc.11.1.18564

[109] Islam F , Gopalan V & Lam AK (2018) RETREG1 (FAM134B): a new player in human diseases: 15 years after the discovery in cancer. J Cell Physiol 233, 4479–4489. 29226326 10.1002/jcp.26384

[110] Islam F , Gopalan V , Wahab R , Smith RA , Qiao B & Lam AK (2017) Stage dependent expression and tumor suppressive function of FAM134B (JK1) in colon cancer. Mol Carcinog 56, 238–249. 27120410 10.1002/mc.22488

[111] Viswanath P , Radoul M , Izquierdo-Garcia JL , Ong WQ , Luchman HA , Cairncross JG , Huang B , Pieper RO , Phillips JJ & Ronen SM (2018) 2-Hydroxyglutarate-mediated autophagy of the endoplasmic reticulum leads to an unusual downregulation of phospholipid biosynthesis in mutant IDH1 gliomas. Cancer Res 78, 2290–2304. 29358170 10.1158/0008-5472.CAN-17-2926 PMC5932252

[112] Bergmann TJ , Fumagalli F , Loi M & Molinari M (2017) Role of SEC62 in ER maintenance: a link with ER stress tolerance in SEC62-overexpressing tumors? Mol Cell Oncol 4, e1264351. 28401179 10.1080/23723556.2016.1264351 PMC5383369

[113] Linxweiler M , Schick B & Zimmermann R (2017) Let's talk about Secs: Sec61, Sec62 and Sec63 in signal transduction, oncology and personalized medicine. Signal Transduct Target Ther 2, 17002. 29263911 10.1038/sigtrans.2017.2 PMC5661625

[114] Peng Y , Shapiro SL , Banduseela VC , Dieterich IA , Hewitt KJ , Bresnick EH , Kong G , Zhang J , Schueler KL , Keller MP , et al. (2018) Increased transport of acetyl-CoA into the endoplasmic reticulum causes a progeria-like phenotype. Aging Cell 17, e12820. 30051577 10.1111/acel.12820 PMC6156544

